# Mechanical Behavior of 3D PolyJet-Printed Nylon 66/Photopolymer Textile Laminates

**DOI:** 10.3390/polym18172136

**Published:** 2026-09-02

**Authors:** Izabela Ciesielska-Wrobel

**Affiliations:** Department of Textiles, Fashion Merchandising and Design, College of Business, University of Rhode Island, 55 Lower College Road, Kingston, RI 02881, USA; iciewrobel@uri.edu

**Keywords:** 3D printing, polyjet printing, 3D printed on textiles, direct-to-textile additive manufacturing, Nylon 66, interlock-knitted fabric, photopolymer, laminate, tensile behavior, accelerated weathering, nonlinear stress–strain behavior, strain stiffening

## Abstract

Large-area three-dimensional polyjet printing (3DPP) of continuous photopolymer laminates directly onto knitted fabrics provides a potential route toward technical textile applications beyond localized decorative features. This study investigated acrylic photosensitive resin (APR) laminates measuring 330 × 432 mm deposited onto a Nylon 66 interlock-knitted fabric. 12- and 20-layer laminates were produced in one-sided and two-sided configurations, and their morphology and tensile behavior were evaluated in the wale and course directions before and after 20 h of accelerated xenon-arc weathering. Scanning electron microscopy (SEM) showed a continuous external APR layer with localized resin entry into inter-yarn and inter-filament spaces, indicating a form-fitting physical connection between the resin and the knitted structure; interfacial strength was not measured. Before weathering, the 20-layer two-sided (20L-2S) architecture exhibited the highest maximum engineering stress, reaching 13.11 ± 0.37 MPa in the wale direction and 7.44 ± 0.28 MPa in the course direction. In contrast, the 20-layer one-sided (20L-1S) architecture retained substantially greater extensibility, reaching maximum engineering strains of 183.94 ± 2.45% and 217.34 ± 2.88% in the wale and course directions, respectively. Thus, two-sided printing maximized load-bearing capacity, whereas one-sided 20-layer printing provided a better balance between reinforcement and preservation of the large-strain response of the knitted substrate. Accelerated weathering reduced the maximum engineering stress of fabric-supported laminates by 5.4–18.7% and maximum engineering strain by 2.1–21.1%, depending on architecture and loading direction. Unsupported APR laminates exhibited 59.9–77.2% increases in maximum engineering stress after exposure, without a corresponding increase in strain capacity. The contrasting response is consistent with APR post-curing or stiffening combined with reduced Nylon 66 extensibility, although chemical and interface-specific tests are required to distinguish these mechanisms. The results demonstrate that continuous 3DPP can produce mechanically integrated textile–photopolymer laminates with tunable strength–extensibility relationships relevant to flexible technical textile structures.

## 1. Introduction

**Figure 1:**
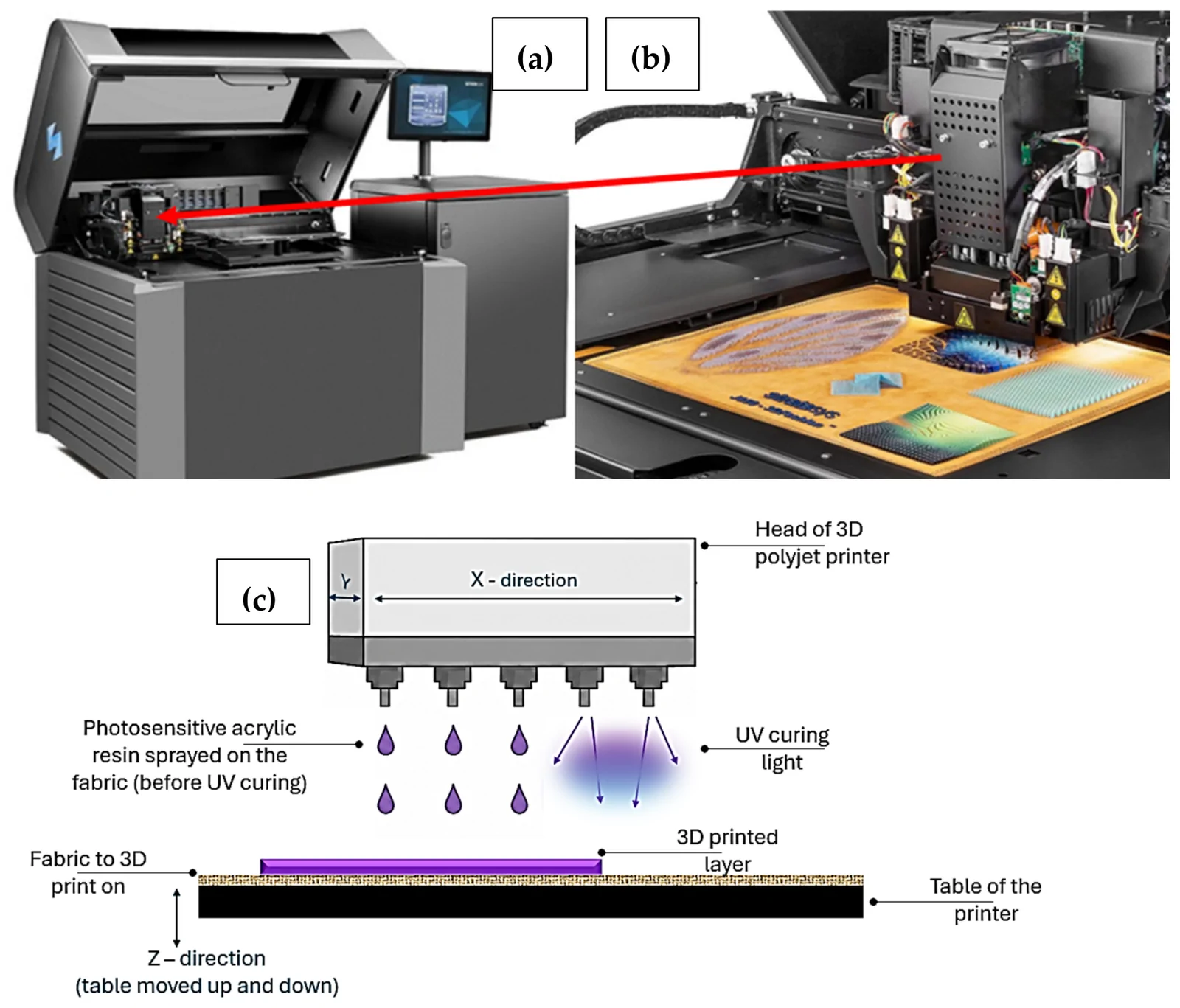
PolyJet printing on textiles: (**a**) printer, (**b**) direct-to-fabric printing system [18], and (**c**) droplet deposition with in situ UV curing.

Three-dimensional printing (3DP), or additive manufacturing (AM), has traditionally been explored and used to produce components for automotive [1], aerospace [2,3], wearables [4], and many types of consumer products, including parts of shoes [5] and headsets, but also educational models, especially for medical studies [6], including dental models and braces [7], but also figurines, gadgets, and toys [8,9], to name some. ISO/ASTM 52900 classifies AM into seven process categories. These are binder jetting, directed energy deposition, material extrusion, material jetting, powder bed fusion, sheet lamination, and vat photopolymerization. These categories distinguish AM technologies by how the feedstock is deposited, joined, or solidified; thus, polyjet differs from processes such as material extrusion, powder fusion, or photopolymerization within a resin vat. PolyJet technology is a form of material jetting in which droplets of the build material are selectively deposited and photopolymerized layer by layer [10,11]. Textile-integrated 3DP has also attracted the fashion industry and designers [12,13,14]. Fashion designer Iris Van Herpen has created both fully 3D-printed garments and structures printed directly on textiles [14,15,16]. Related design work has highlighted the functional and expressive possibilities of combining printed polymers with fabric substrates [17]. Three-dimensional polyjet printing (3DPP) technology allows printing on both conventional fabrics and meshes to form clothing on demand, haute couture, and fashion accessories, as proven by Van Herpen and Karim Rashid in the last decade. The 3DPP allows the use of up to 600,000 colors and combines opaque or translucent photopolymers with different Shore hardness. Functional examples include small, periodically spaced Braille domes printed directly on fabrics [18]. Figure 1 illustrates direct-to-textile 3DPP. The fabric is fixed to the build platform, droplets of UV-curable APR are deposited according to a digital design, and each layer is cured in situ before the next layer is deposited [17,18].

### 1.1. Printing on Textiles for Functional and Technical Applications

Although direct 3DPP on textiles has attracted some interest, its wider, non-artistic applications remain limited. Published studies have primarily examined printing feasibility, polymer–textile adhesion, surface morphology, and durability after washing, abrasion, or light exposure. Comparatively little attention has been given to continuous, large-area 3DPP laminates that function as mechanically active components of textile–polymer structures. Most published studies have focused on small-sized 3DPP on woven fabrics, leaving a relative dearth of information on knitted fabrics, which exhibit different stretch, drape, and porosity characteristics [19]. Knitted fabrics differ fundamentally from woven substrates because their deformation is initially governed by loop rotation, loop opening, yarn bending, and interloop sliding, followed by increasing yarn alignment at higher strain. Consequently, the response of a printed knitted laminate cannot be inferred directly from results obtained for relatively dimensionally stable woven fabrics. Prior studies have shown that adhesion between the printed polymer and the textile depends on fiber type, surface morphology, textile architecture, and printing parameters, including temperature, printing speed, and layer thickness [19,20,21]. Using a Stratasys Connex 350 printer and FullCure photopolymer materials, the researchers printed polymer structures directly onto several textile substrates and reported adhesion forces of approximately 30–35 N. Their study established that 3DPP can be used to produce polymer–textile composite structures, while also identifying several practical challenges, including resin flow through highly porous fabrics and fixation of textile substrates on the build platform. In a different study, Braille domes were printed onto cotton and polyester woven fabrics, and the resulting materials were evaluated for washing resistance, colorfastness to light, tensile behavior, and abrasion resistance. The results showed that the resin remained attached to the textile substrates after washing; however, the printed structures were sensitive to light exposure and abrasion. Tensile testing also indicated that the presence of printed resin features reduced the strength and elongation of the fabrics. This work demonstrated that the 3DPP can modify textile performance, although the localized, periodically repeated design was evaluated primarily as a surface feature rather than as a continuous mechanical laminate [18]. More recently, studies using medical polyjet resins such as MED610 have investigated the influence of textile substrates on adhesion and surface morphology [21]. This direction is particularly relevant because MED610 is a biocompatible resin, suggesting potential future applications in medical and biomedical textile composites. However, the studies have focused mainly on material compatibility, adhesion, surface roughness, hydrophobicity, porosity, and morphology rather than on the development and validation of complete technical textile prototypes [22]. Overall, the current literature on 3DPP on textiles demonstrates that UV-curable photopolymer resins can be successfully deposited onto selected textile substrates and that adhesion, substrate morphology, resin penetration, and environmental durability are among the key challenges [16,17,18,19,20,21,22,23,24]. Other research investigated composites made by laminating 3D-printed re-entrant thermoplastic polyurethane patterns onto aramid knitted fabric. The laminated structures showed improved mechanical performance and energy absorption compared with the unmodified knit, indicating their potential for protective textile applications. However, the polymer patterns were printed separately and then bonded to the fabric rather than printed directly onto the textile substrate [25]. In general, most published relevant studies remain focused on printability, adhesion testing, surface morphology, or localized surface features [19,20,21,22,23,24,25].

Material-extrusion studies have quantified the effects of printing temperature, speed, stand-off distance, pretreatment, and fabric structure on peel resistance [12], while embedded-filament work on knitted textiles showed that loop geometry and the incorporation method affect bonding and deformation [13]. Vat photopolymerization on textile fabrics has also used lower-viscosity resin infiltration and evaluated composite bending behavior [14]. These studies represent important progress but use printing processes, specimen dimensions, and loading modes that differ from continuous large-area polyjet printing. Evidence therefore remains limited for continuous polyjet laminates printed directly on highly extensible knitted fabrics and evaluated across architecture, wale/course direction, and accelerated weathering.

### 1.2. Purpose of the Study

Research remains scarce on continuous, large-area 3DPP laminates deposited directly onto textiles. Therefore, the purpose of this study was to investigate continuous 3DPP laminates using APR, measuring approximately 330 × 432 mm, produced on knitted Nylon 66 fabric, and to determine how laminate architecture influences the mechanical behavior of the resulting textile–polymer system. Laminates comprising different numbers of printed layers were applied either to one side or to both sides of the knitted substrate. The effects of printed-layer configuration, one- versus two-sided application, fabric direction (wale and course), and accelerated UV exposure (aging) on tensile behavior were evaluated. The broader motivation for this work was to explore the potential of 3DPP textile–polymer laminates for technical textile applications. It is hypothesized that a continuous large-area APR layer may reduce the openness of the knitted structure and potentially enhance surface protection or barrier functionality, while the knitted Nylon 66 substrate may contribute flexibility and extensibility. Such material combinations could be relevant to flexible barriers, protective covers, inflatable structures, and other technical textile applications requiring a balance of strength, flexibility, stretchability, durability, and surface protection. The tensile tests quantified the global response of the complete textile–APR architecture. They did not measure resin-to-fabric peel strength or interfacial shear strength.

## 2. Materials and Methods

### 2.1. Initial Three-Dimensional Polyjet Printing (3DP)—Exploratory Study

**Figure 2:**
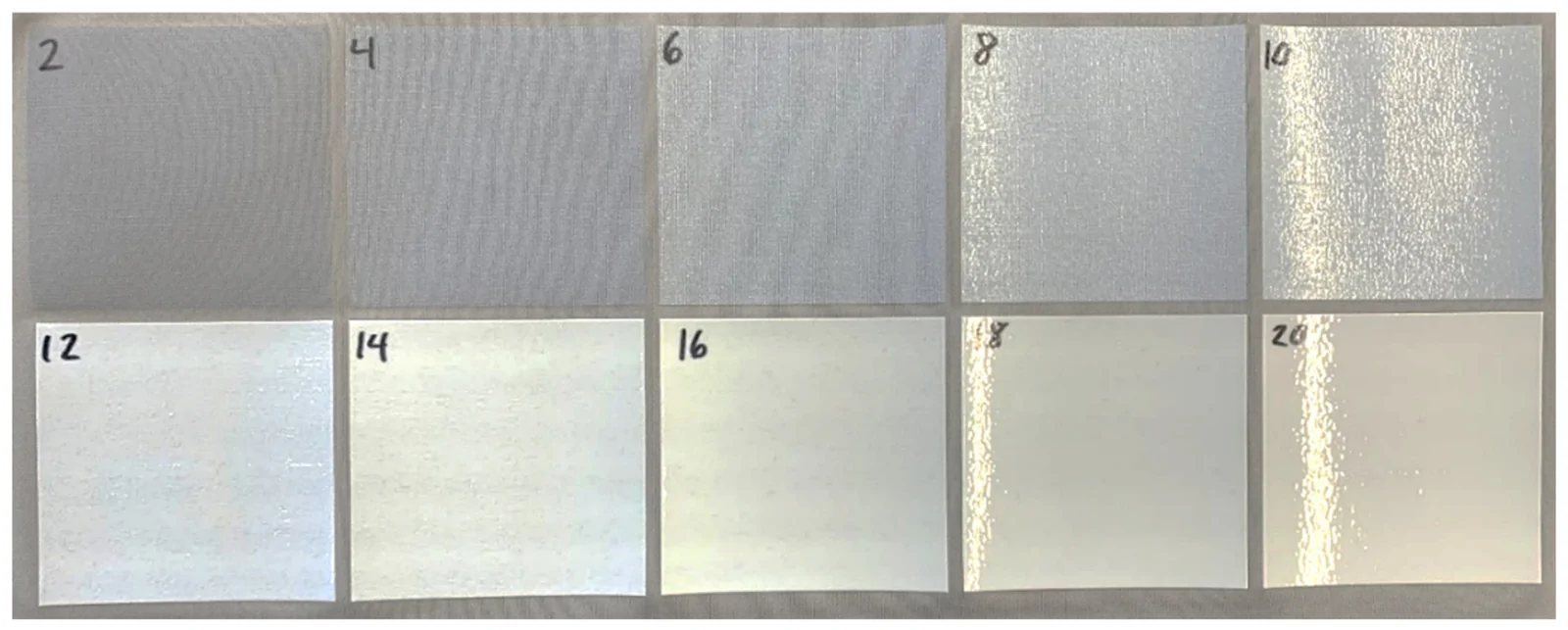
Exploratory 3DPP specimens with 2–20 APR layers on Nylon 66 interlock fabric. The 8 × 8 cm squares shown in Figure 2 correspond to the enlarged images presented in Figure 3.

An initial exploratory study was conducted to determine suitable printed-layer configurations for producing larger-area textile–APR laminates. Square specimens measuring 8 × 8 cm were prepared by Stratasys, Ltd., Eden Prairie, MN, USA, using progressively increasing layer numbers ranging from 2 to 20 layers (Figure 2). The specimens, printed on a grey double-knit interlock Nylon 66 fabric, were visually examined from both the printed and reverse sides to assess surface coverage and resin penetration through the fabric. The specimens were printed on a grey double-knit interlock Nylon 66 fabric used only for the exploratory study and distinct from the white Nylon 66 fabric used in the main experiment. This preliminary study was conducted to identify suitable layer configurations for producing larger-area laminates. Specimens with fewer than 12 layers showed incomplete surface coverage, whereas the 12-layer configuration was the first to form a continuous surface without visible pores in the laminate. Increasing the number of layers to 20 produced a thicker laminate with more complete coverage. Accordingly, the 12- and 20-layer configurations were selected for the main study. Based on the difference between the thicknesses of the printed and unprinted fabrics, the estimated thickness of a single deposited layer was 0.022 mm ± 2%. The APR did not penetrate completely through the fabric, as no resin was visible on the technical back (reverse) of the specimens.

### 2.2. Fabric Used in Three-Dimensional Polyjet Printing (3DPP)

A double stitch, interlock, weft knit, white, 100% Nylon 66 fabric was selected as a substrate for 3DPP. This fabric was purchased at Testfabrics, Inc., West Pittston, PA, USA. It was selected because Nylon 66 is one of the strongest conventional (non-high performance), manufactured raw materials and is traditionally used for conventional and some technical applications, e.g., parachutes, braided, twisted, and circular knitted ropes, and materials in inflatables. The structure of the fabric is presented in Figure 4, where wales (vertical direction) and courses (horizontal direction) are shown. The interlock-knitted fabric was selected as the textile substrate because its double-knit architecture provides a relatively stable and uniform surface for direct 3D deposition while retaining the loop-mediated deformability characteristic of knitted structures. Compared with conventional non-stretch woven fabrics, the knitted construction can accommodate greater deformation through changes in loop geometry, even without incorporating elastomeric yarns.

### 2.3. Acrylic Photosensitive Resin (APR) Used for 3DPP Lamination

The APR used to form the 3DPP laminates on the surface of the knitted Nylon 66 fabrics was VeroEco™Flex developed by Stratasys, Ltd., Eden Prairie, MN, USA. The grey laminate color used in this study was generated from a combination of VeroEco™Flex Cyan [26], VeroEco™Flex Magenta [27], and VeroEco™Flex Yellow [28]. The three resin components had similar basic physical characteristics. The reported density was 1.079 g/cm^3^ for VeroEco™Flex Cyan, 1.077 g/cm^3^ for VeroEco™Flex Magenta, and 1.075 g/cm^3^ for VeroEco™Flex Yellow. According to the corresponding Safety Data Sheets (SDSs), all three materials are liquids and are insoluble in water. The chemical composition of the resin systems was only partially disclosed by the manufacturer in the available Safety Data Sheets [26,27,28]. A substantial proportion of each formulation was represented by constituents identified only as proprietary. The SDSs listed six proprietary constituent entries for both VeroEco™Flex Cyan and VeroEco™Flex Magenta, while VeroEco™Flex Yellow contained seven proprietary constituent entries. The disclosed ingredients common to all three formulations were camphene (CAS 79-92-5), 2,6-bis(1,1-dimethylethyl)-4-methylphenol (CAS 128-37-0), glycerol, propoxylated, esters with acrylic acid (CAS 52408-84-1), and 1,7,7-trimethyltricyclo [2.2.1.0^2,6^]heptane (CAS 508-32-7), each reported at 0.1–0.3 wt.%. The exact percentage or concentration of the composition was withheld as a trade secret.

### 2.4. Main Experiment—3DPP on Textiles

**Figure 3:**
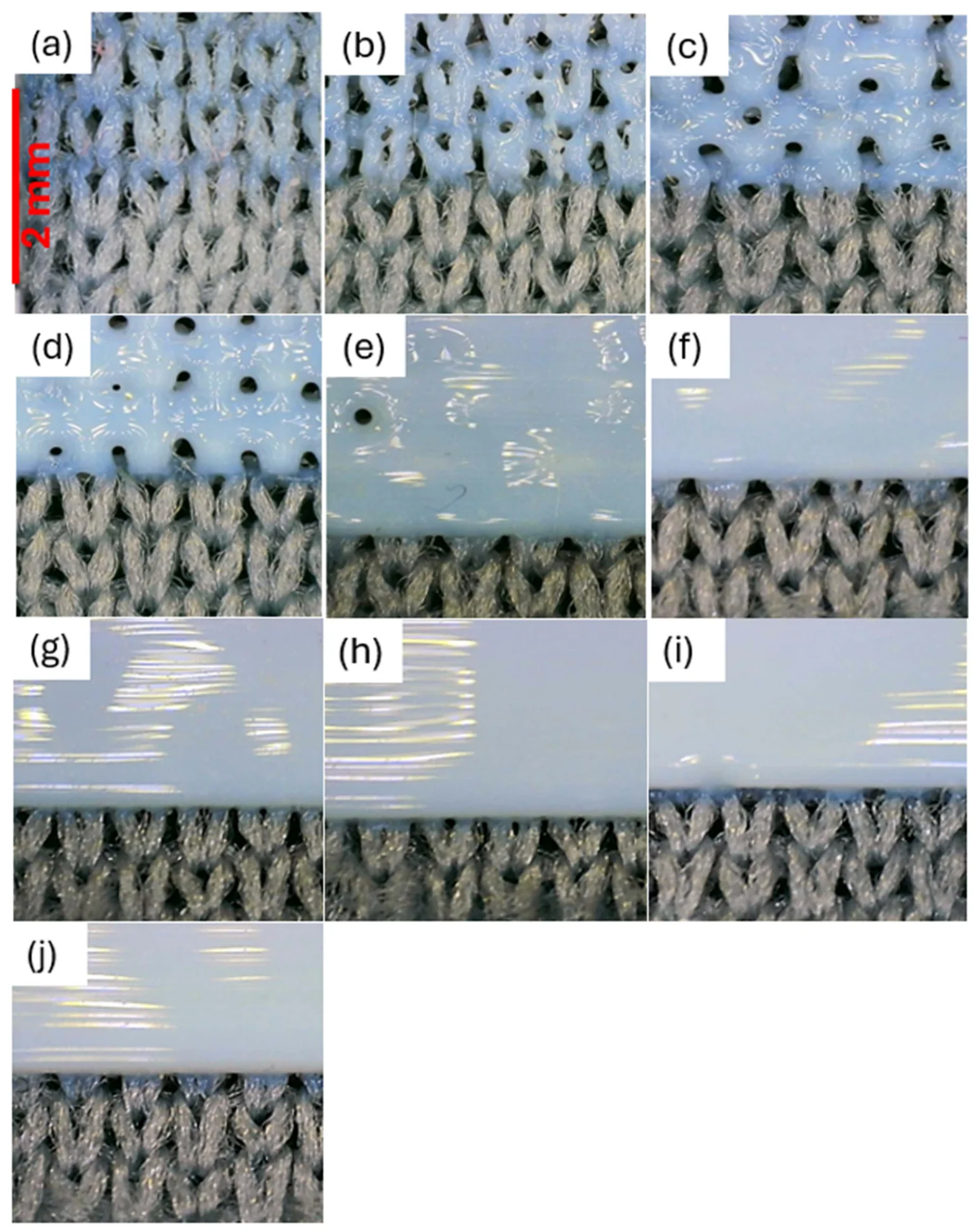
Enlarged exploratory specimens with increasing APR layers: (**a**) 2, (**b**) 4, (**c**) 6, (**d**) 8, (**e**) 10, (**f**) 12, (**g**) 14, (**h**) 16, (**i**) 18, and (**j**) 20.

**Figure 4:**
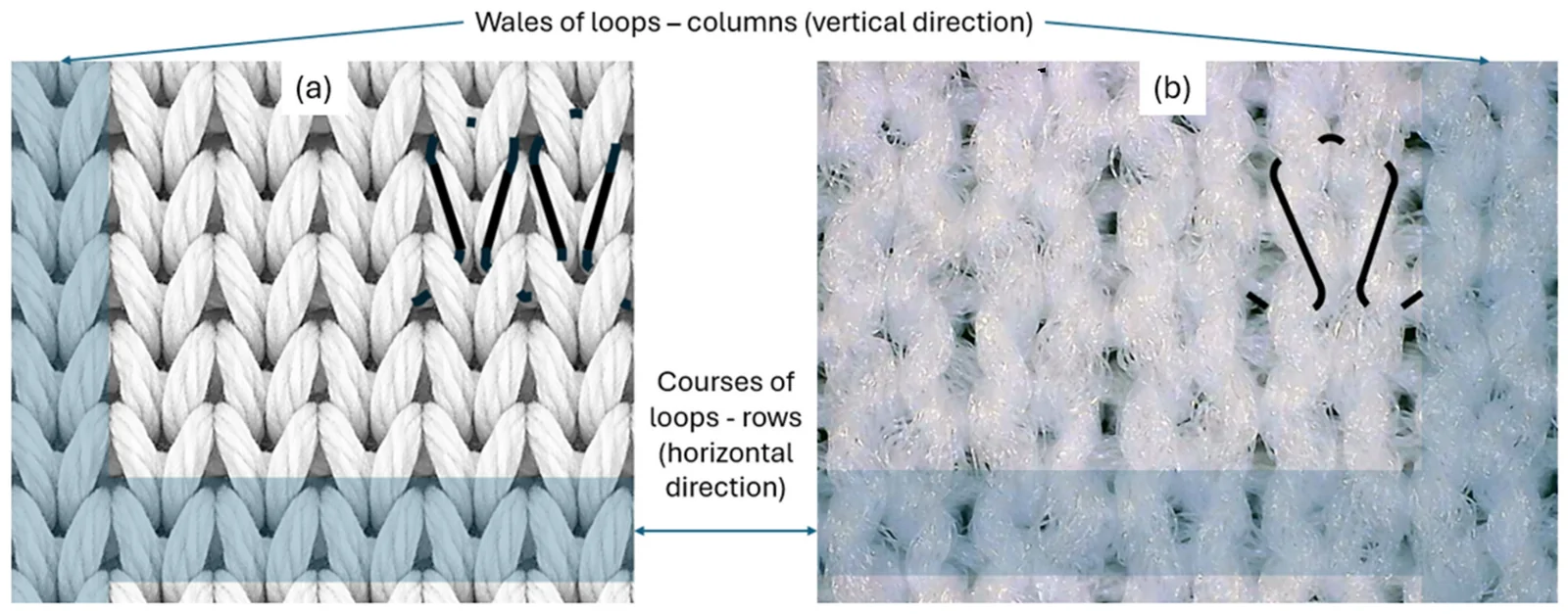
Nylon 66 interlock knit: (**a**) idealized technical face and (**b**) observed loop structure; wale and course directions are indicated.

Based on the exploratory study, 12-layer and 20-layer configurations were selected for the main experiment. The 12-layer configuration represented the lowest number of printed layers that produced a visually continuous laminate without apparent open pores on the surface, see Figure 3f. The 20-layer configuration was selected because it represented the maximum layer number evaluated during the preliminary screening and produced the greatest degree of surface coverage and pore closure, see Figure 3j. The comparison between 12 and 20 layers therefore enabled assessment of how increasing the amount and thickness of the deposited APR influenced laminate mechanical performance. Table 1 lists samples that were prepared, analyzed, and tested.

The laminates were produced by Stratasys, Ltd., using a Stratasys J850 TechStyle system. According to the manufacturer’s specifications, the equipment operates within an ambient temperature range of 18–25 °C and a relative-humidity range of 30–70% under non-condensing conditions. The high-speed module used to print the laminates provides a nominal layer resolution of 27 μm, and the deposited APR is cured during printing using two integrated, independently controlled UV LED units [16,17,18]. The surface temperature of the fabric was not measured during printing. This job-specific UV irradiance, print-head speed, and inter-pass UV exposure duration were also not retained in the available production record and were not varied as experimental factors.

### 2.5. Basic Characteristics of Fabrics and Laminates

Fabric and laminate characteristics were measured according to American Society for Testing and Materials (ASTM) standards for fabric weight [29], fabric count [30], and thickness [31], and are presented in Table 2. Specimens were conditioned for 24 h at 21 °C and 65% relative humidity before measurement. Printed panels received from Stratasys Ltd. were conditioned under the same conditions for at least 24 h before testing. Each printed area measured 13 × 17 in. (330.2 × 431.8 mm).

The unsupported laminate-only specimens were produced as independent reference structures and did not represent APR physically separated from the corresponding fabric-supported laminates. The combined thickness of a supported architecture includes the external APR region, the knitted substrate, and localized resin entry into textile voids; therefore, constituent thicknesses cannot be subtracted to obtain a valid penetration depth. Resin penetration was assessed qualitatively from representative SEM cross-sections. A penetration depth expressed as a percentage of total fabric thickness was not calculated because the manually cut or fractured, non-polished sections did not provide a calibrated, normal-to-surface full-thickness plane suitable for statistically defensible measurement.

#### 2.5.1. Scanning Electron Microscopy (SEM)

Scanning electron microscopy (SEM) was used to examine both the surface morphology and cross-sectional structure of the 3DPP laminates and their interaction with the Nylon 66 knitted substrate. Surface images were obtained to evaluate the continuity and uniformity of the printed layer and the extent of coverage of the knitted fabric. Cross-sectional images were used to examine the APR–textile interface, including penetration of the APR into the inter-yarn and inter-filament spaces and the physical configuration of the laminate within the textile structure. Cross-sections were prepared by manual cutting or fracture rather than metallographic polishing. SEM specimens were representative, previously untested pieces and were not removed from tensile-fracture zones. Consequently, the micrographs cannot identify post-tensile failure as delamination, yarn pull-out, or cracking of the APR matrix. The images were acquired using a secondary-electron detector at an accelerating voltage of 5.0 kV, a working distance of approximately 7.9 mm, and a 30 µm aperture. Images were recorded at magnifications of 100×, 150×, and 500×.

#### 2.5.2. Accelerated Weathering

Accelerated xenon-arc weathering was conducted to evaluate the effect of 20 h of accelerated xenon-arc exposure on the appearance and mechanical behavior of the unprinted Nylon 66 knitted fabric and the Nylon 66 fabrics with 3DPP laminates, as well as the laminates only. The exposure procedure was based on AATCC TM169-2020, Weather Resistance of Textiles: Xenon Lamp Exposure. Testing was performed using a Q-SUN Xe-3-HS xenon-arc weathering chamber (Q-Lab Corporation, Westlake, OH, USA) [32]. The specimens were exposed continuously for 20 h at a spectral irradiance of 1.10 W·m^−2^·nm^−1^ controlled at 420 nm. The specimens were mounted flat in the exposure chamber. Regardless of whether the samples were one-sided or two-sided laminate structures, only one surface was oriented toward the xenon-arc light source and therefore exposed to the accelerated weathering. For the one-sided structures, the 3DPP-laminated surface was oriented toward the xenon-arc light source. Following exposure, the samples were visually examined for changes in appearance, including discoloration, yellowing, surface cracking, embrittlement, laminate damage, and local separation of the printed layer from the knitted substrate. The unexposed and weathered specimens were subsequently subjected to tensile testing to determine the effect of 20 h of accelerated weathering on their stress–strain behavior.

#### 2.5.3. Tensile Properties

Tensile testing used a strip method based on ASTM D5035 [33] and an ADMET eXpert 7601 universal testing system controlled by MTestQuattro software (Version 7.03.01), which acquired and recorded force and crosshead-displacement data. Specimens were tested at a crosshead speed of 254 mm/min (10 in/min). Specimen dimensions were 50 mm wide and 150 mm long, and specimens were prepared in the wale and course directions and tested using an initial gauge length of 100 mm. Engineering strain was calculated from crosshead displacement relative to the initial gauge length. Engineering stress was calculated from the recorded tensile force using the measured specimen width and the total measured thickness of the tested structure, including both the knitted Nylon 66 substrate and the printed APR laminate where applicable. Three specimens were tested for each material architecture, direction, and exposure condition. Individual curves were inspected before calculation of group statistics. Mean stress–strain curves were generated by interpolation onto a common strain grid, and the shaded regions represent ±1 sample standard deviation (SD) over the strain range for which at least two specimens remained. Neither a conventional contact extensometer nor a video extensometer was used during this test. These strip tests quantify the global tensile response of the complete architecture. No peel, lap-shear, or other interface-specific test was performed, so the data are not interpreted as resin-to-fabric peel strength or interfacial shear strength.

## 3. Results

**Figure 5:**
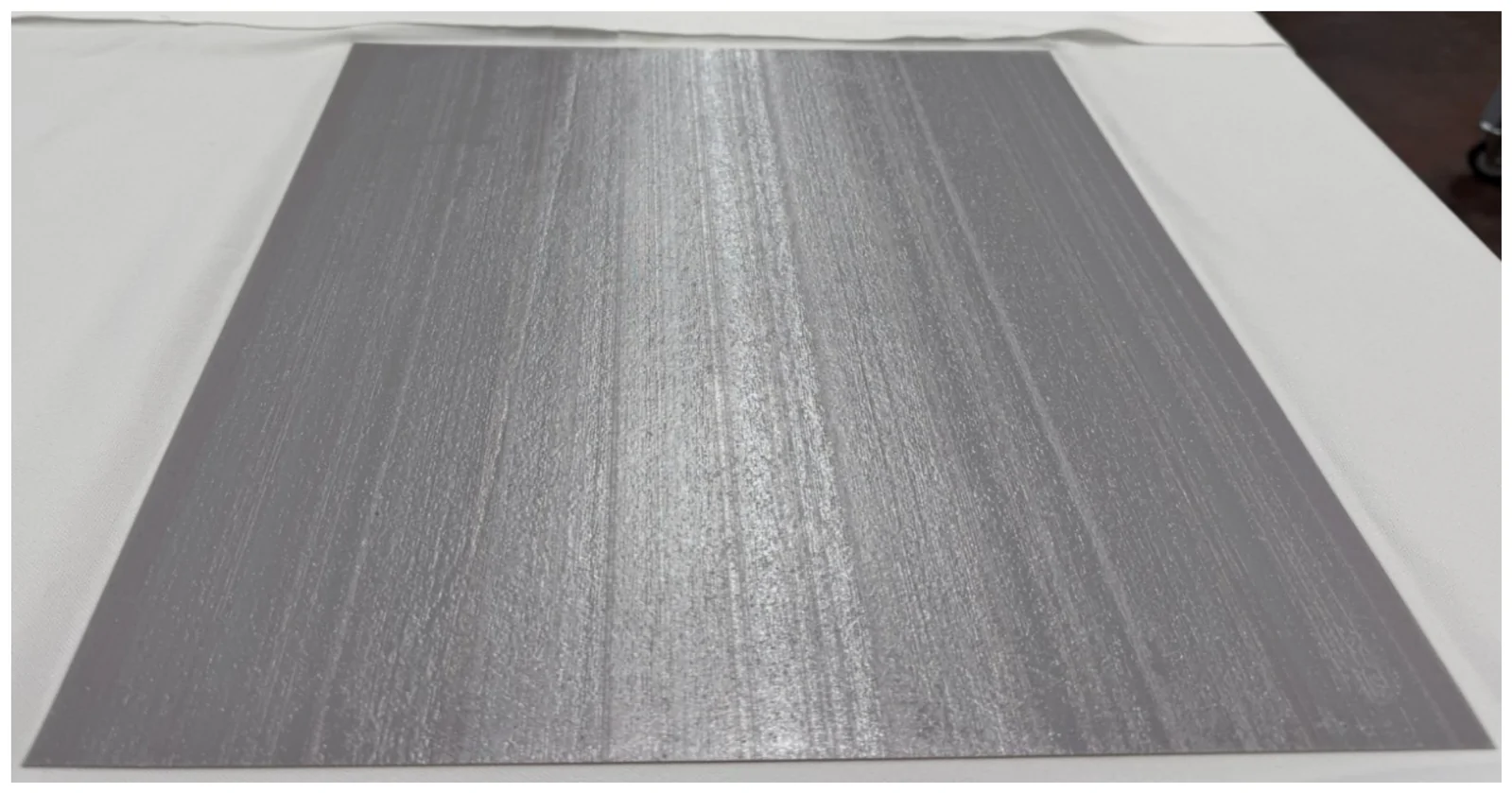
Representative 330.2 × 431.8 mm continuous APR laminate printed on knitted fabric.

Figure 5 presents a large-area 3DPP laminate measuring 330.2 × 431.8 mm. The APR formed a continuous laminate across the printed area. Parallel surface striations were visible on all panels. These striations were not intentional design features but resulted from the printing path used during APR deposition. The striations were oriented parallel to the longer dimension of the panel, corresponding to successive jetting and curing passes along the 431.8 mm edge. These process-related variations likely contributed to the measured variability in mass per unit area and thickness (see Table 2). Although such process-related surface patterning is undesirable from the standpoint of surface uniformity, it can occur during large-area printing and provides a visible indication of the printing direction. SEM observation also confirmed the striations.

### 3.1. Scanning Electron Microscopy (SEM)

**Figure 6:**
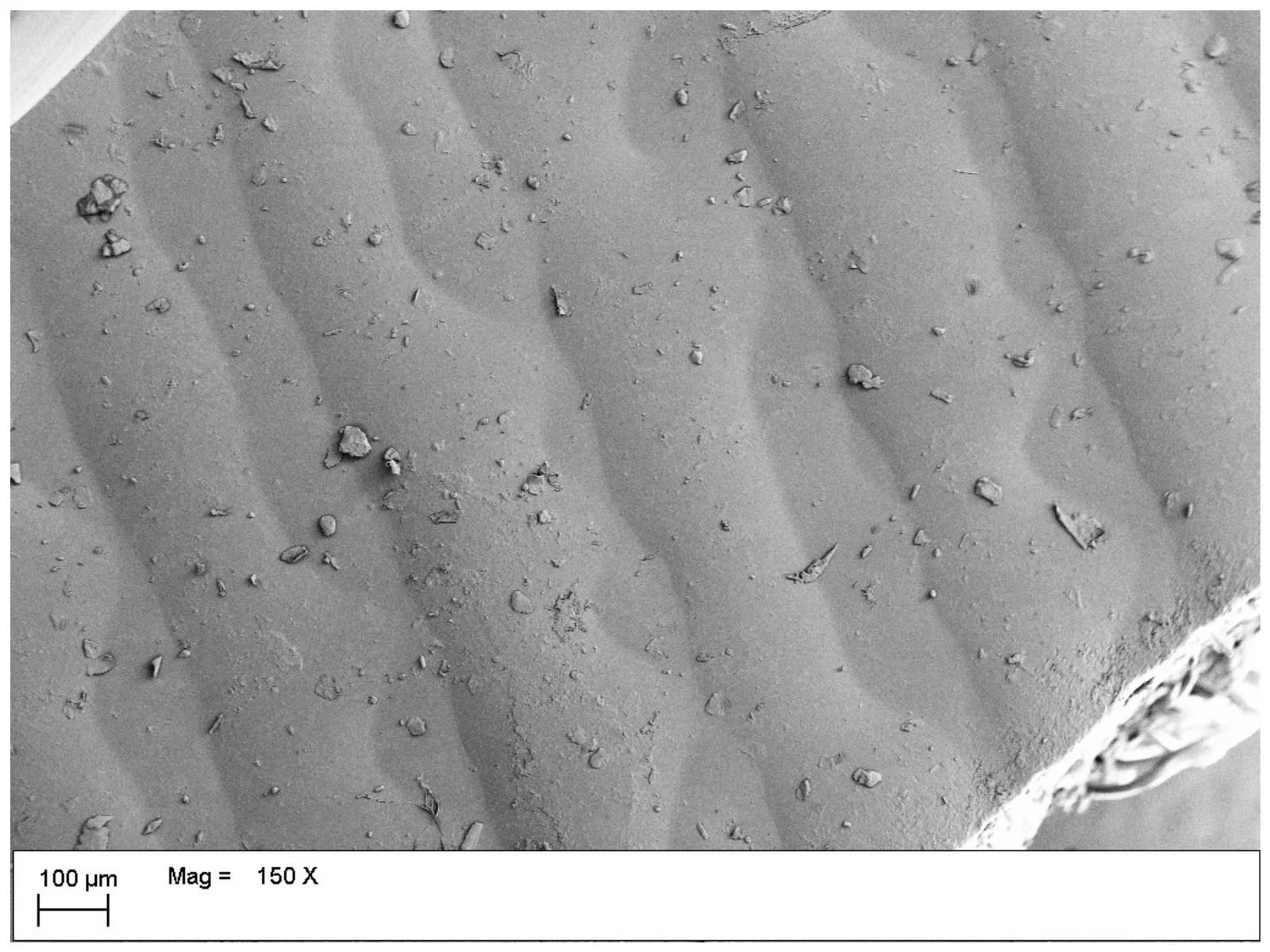
Surface SEM image of a representative 3DPP laminate showing a continuous APR surface with parallel printing-related striations and scattered irregular surface features. Magnification: 150×; scale bar: 100 µm.

**Figure 7:**
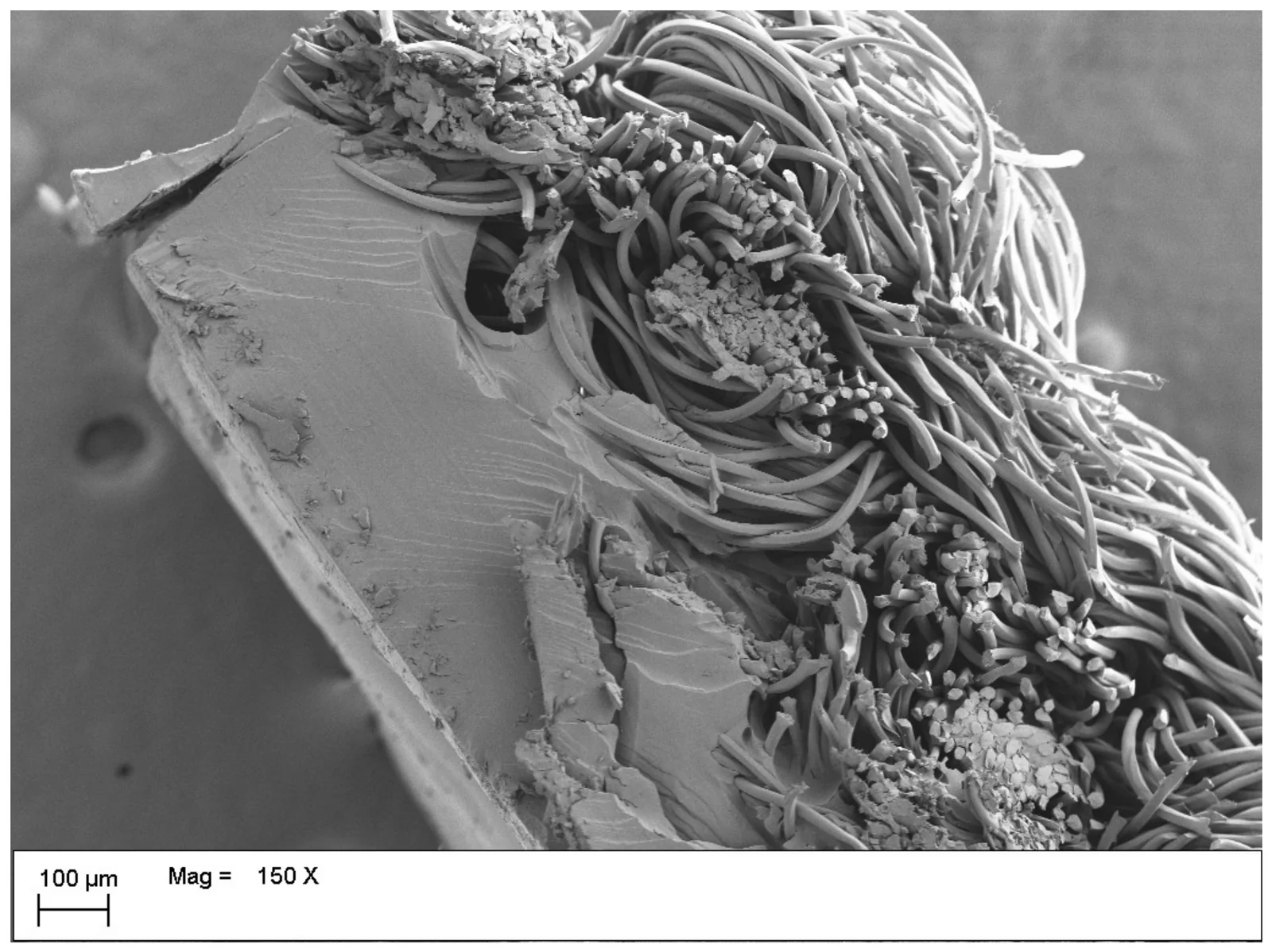
12L-1S cross-section showing external APR and localized entry into textile voids (150×; scale bar 100 µm).

**Figure 8:**
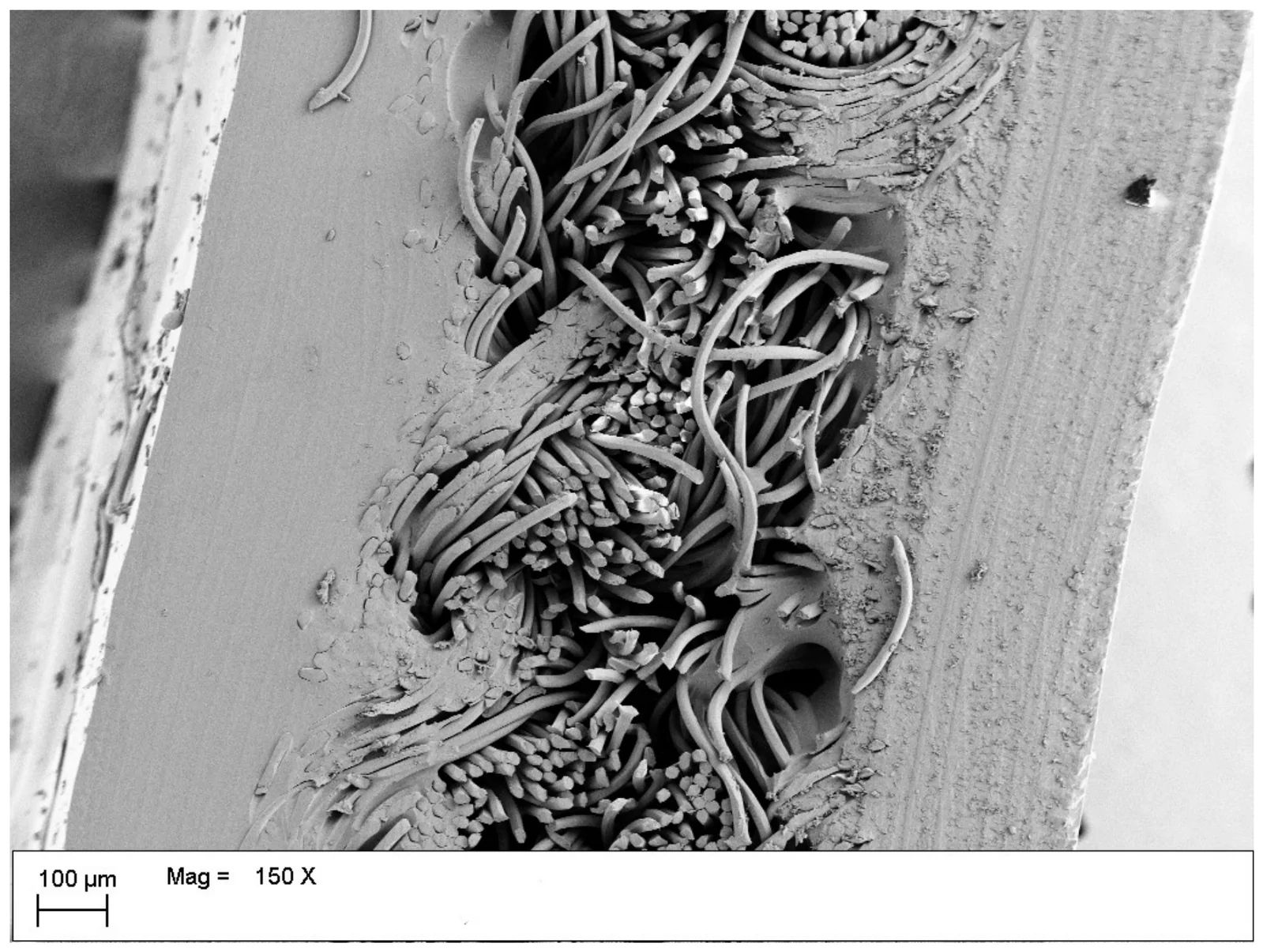
Two-sided (12L-2S) cross-section with a central knitted zone and retained voids (150×; scale bar 100 µm).

**Figure 9:**
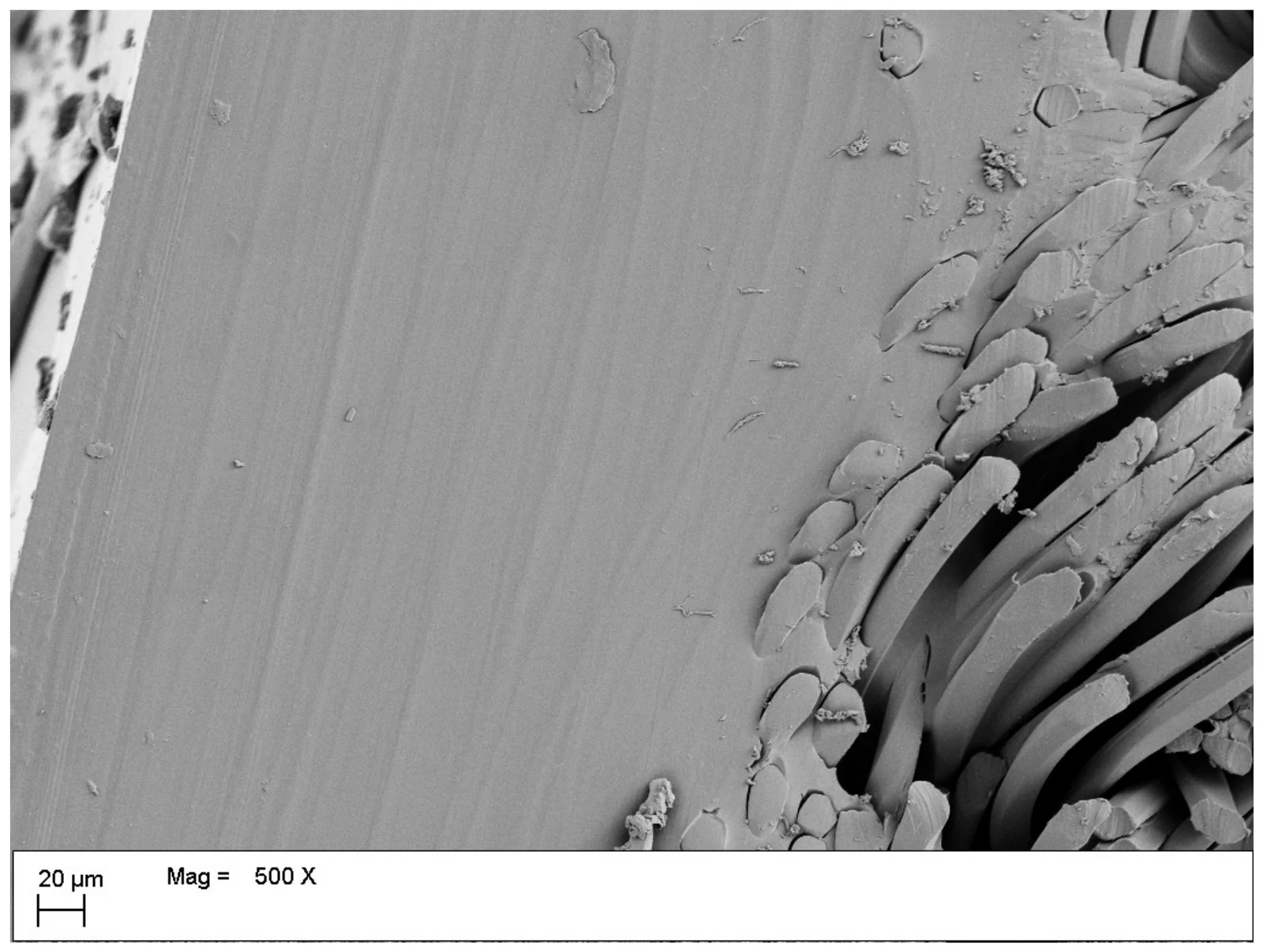
Higher-magnification interface from Figure 8; APR locally surrounds filaments (500×; scale bar 20 µm).

**Figure 10:**
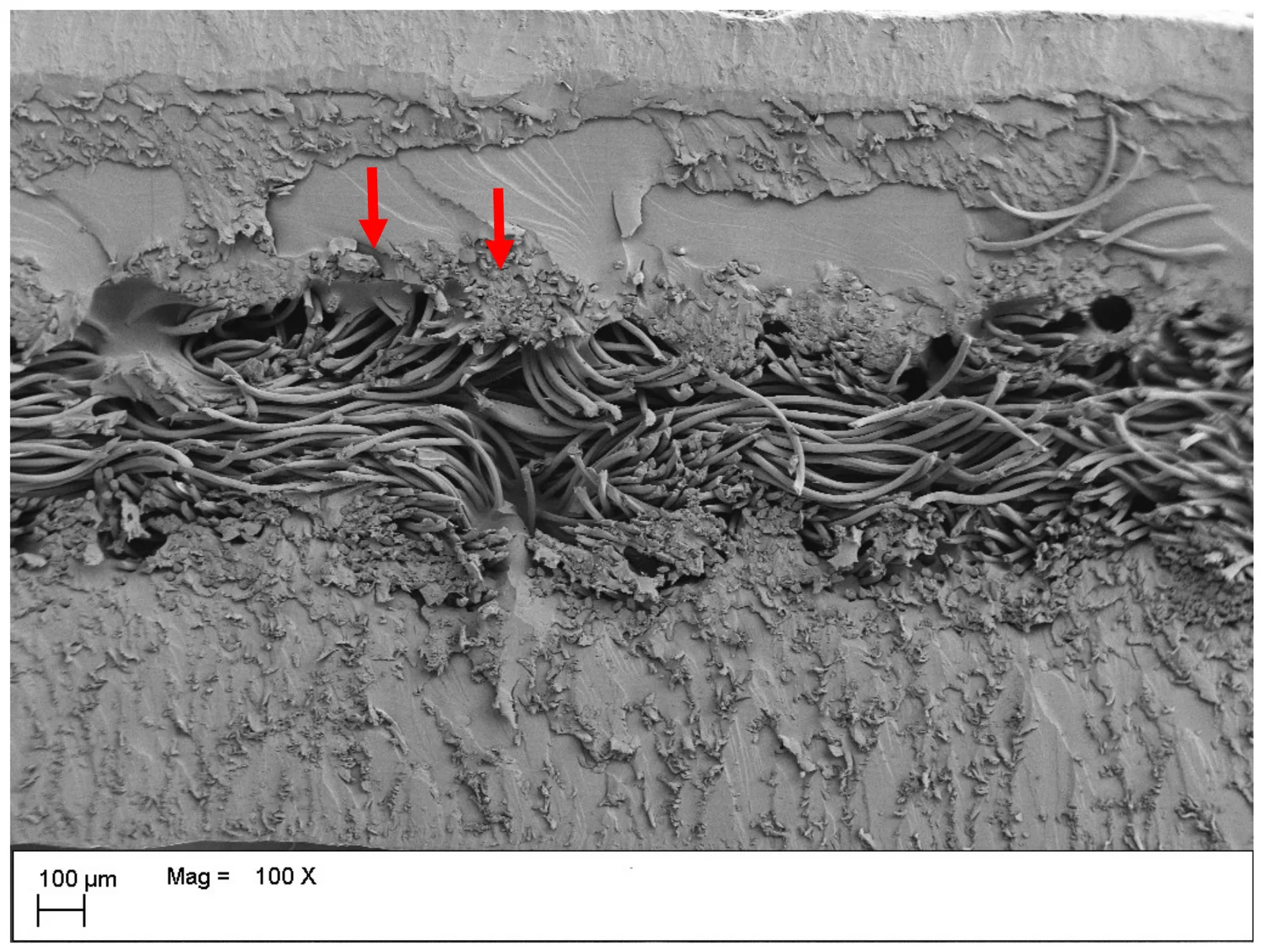
Two-sided (20L-2S) cross-section with heterogeneous APR enclosure of filament bundles (indicated by red arrows) (100×; scale bar 100 µm).

**Figure 11:**
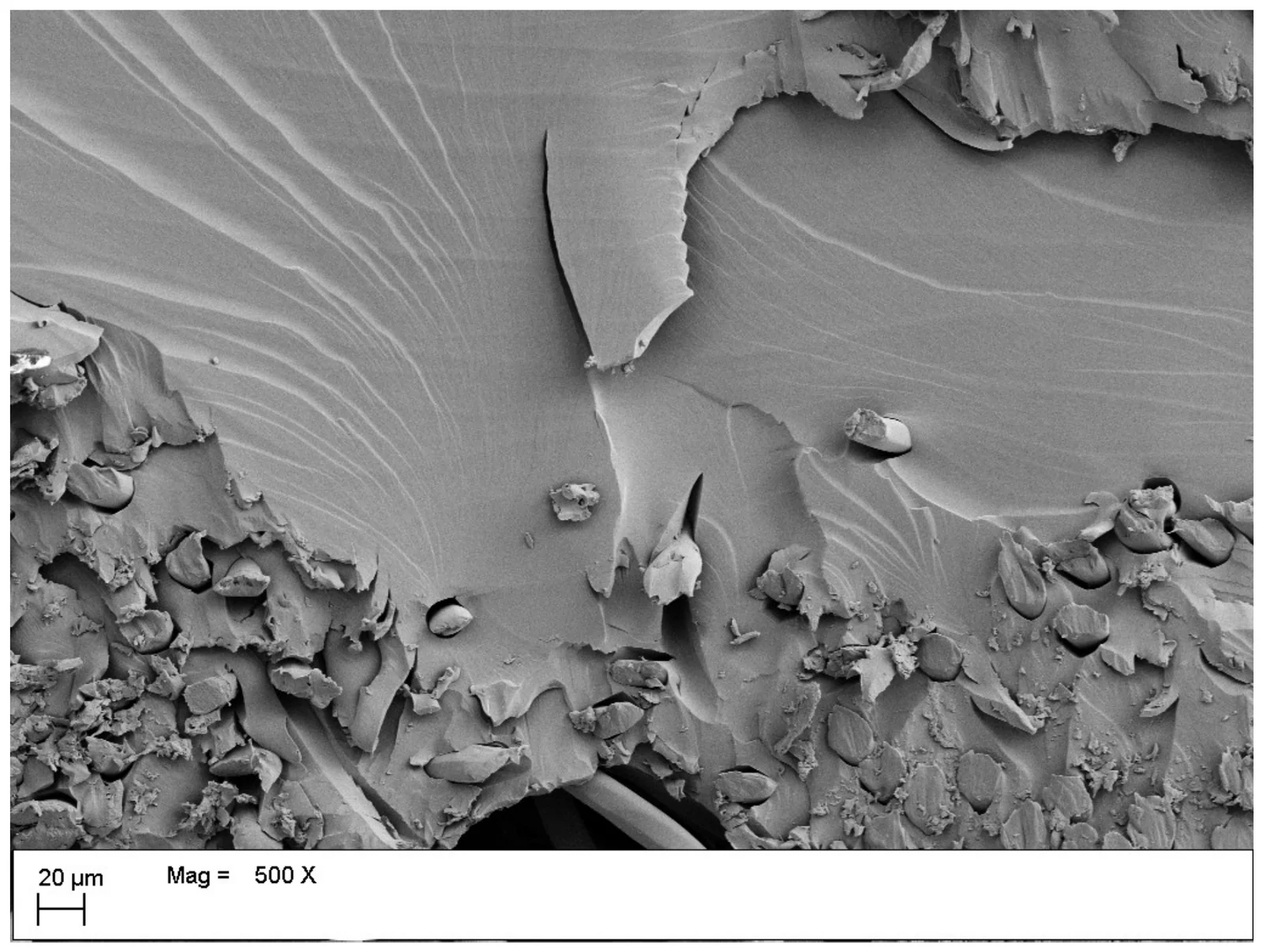
Higher-magnification view of locally APR-enclosed filaments in Figure 10 (500×; scale bar 20 µm).

The representative surface image in Figure 6 shows that the APR formed a largely continuous layer over the textile substrate. Approximately parallel surface undulations were visible and corresponded to the striations observed in the macroscopic panel photograph (see Figure 5). The printed surface was therefore continuous but not completely smooth or uniform. Small, irregular surface features were also distributed across the laminate. Because their chemical composition was not determined, they are described here only as morphological surface features and cannot be conclusively identified as contamination, residual material, or fragments produced during specimen preparation. The cross-sectional image of the 12-layer one-sided laminate in Figure 7 shows a compact APR region aligned with the knitted substrate. The APR extended into spaces between yarns and locally surrounded individual Nylon 66 filaments. However, the penetration was not uniform throughout the textile structure, and some filament bundles remained only partially surrounded by resin. These observations indicate that the laminate was connected to the textile through a combination of surface coverage and localized penetration into the porous knitted structure. The morphology is consistent with potential form-fitting or geometric engagement, but it does not establish interface strength. The two-sided laminate shown in Figure 8 exhibited a different cross-sectional architecture, with the Nylon 66 knitted structure positioned between the APR regions applied to both surfaces. A higher-magnification image in Figure 9 further showed close contact between the APR and individual filaments, as well as partially filled inter-filament spaces and irregular fracture surfaces. The irregular edges and loose fragments visible in Figure 8, Figure 9, Figure 10 and Figure 11 originated from cutting or fracturing during SEM specimen preparation; they are not post-tensile fracture features. Additional cross-sectional images of the 20-layer laminate are presented in Figure 10 and Figure 11, further illustrating localized APR penetration around filament bundles and individual filaments. Across Figure 7, Figure 8, Figure 9, Figure 10 and Figure 11, the common architecture was a continuous external APR region with spatially nonuniform entry into textile voids. The 12-layer one-sided specimen showed localized resin entry from one face, whereas the two-sided specimen contained APR regions on both faces around a central knitted zone that retained open voids. The 20-layer images showed a more substantial external APR region but still did not indicate uniform impregnation through the full fabric thickness. These observations support geometric engagement between APR and the porous knit but do not quantify bond strength or identify the tensile failure mode.

Comparison of Figure 7, Figure 8, Figure 9, Figure 10 and Figure 11 indicates that increasing the number of deposited APR layers and applying the laminate to both sides altered primarily the distribution and extent of the APR-rich regions rather than producing uniform impregnation of the knitted structure. The 12L-1S cross-section in Figure 7 shows a distinct external APR region with localized entry from the printed face and partial enclosure of the adjacent yarn and filament bundles. In the two-sided cross-section shown in Figure 8 and Figure 9, the knitted structure is positioned between APR regions formed on the two surfaces, while open inter-yarn and inter-filament spaces remain within the central textile zone. The 20-layer cross-sections in Figure 10 and Figure 11 show a more substantial external APR region and more extensive local enclosure of filament bundles; nevertheless, resin distribution remains heterogeneous, and unfilled textile voids are still present. Therefore, these structures are more appropriately described as surface laminates with localized resin entry than as fully impregnated textile composites.

These morphological differences provide a plausible structural basis for the tensile behavior. A thicker or two-sided APR architecture can constrain loop rotation and opening over a larger portion of the knitted structure, consistent with the higher mean maximum engineering stress of the 20L-2S specimens. In contrast, the one-sided architecture leaves the opposite face unconstrained and retains more open textile volume, which may contribute to the greater extensibility observed for the 20L-1S specimens. Transitions between APR-rich regions, partially enclosed filament bundles, and unfilled voids may also create local stiffness gradients and concentrations of bending and shear during loop deformation. These regions provide plausible sites for the initiation of sequential APR cracking and the stepwise stress reductions observed during tensile testing. However, because the SEM images were obtained from manually prepared, previously untested cross-sections, they do not directly identify crack-initiation sites, quantify interfacial strength, or establish the tensile failure mechanism. Additional SEM images are available in the Supplementary Materials. Following 20 h of accelerated xenon-arc exposure, visible fading of the grey APR surface was observed. Because color change was not quantified instrumentally, this observation is reported qualitatively.

### 3.2. Engineering Stress–Strain Behavior

The tensile behavior of the unprinted Nylon 66 knit, the fabric-supported 3DPP laminates, and the unsupported laminate-only specimens were evaluated before and after accelerated xenon-arc exposure.

**Figure 12:**
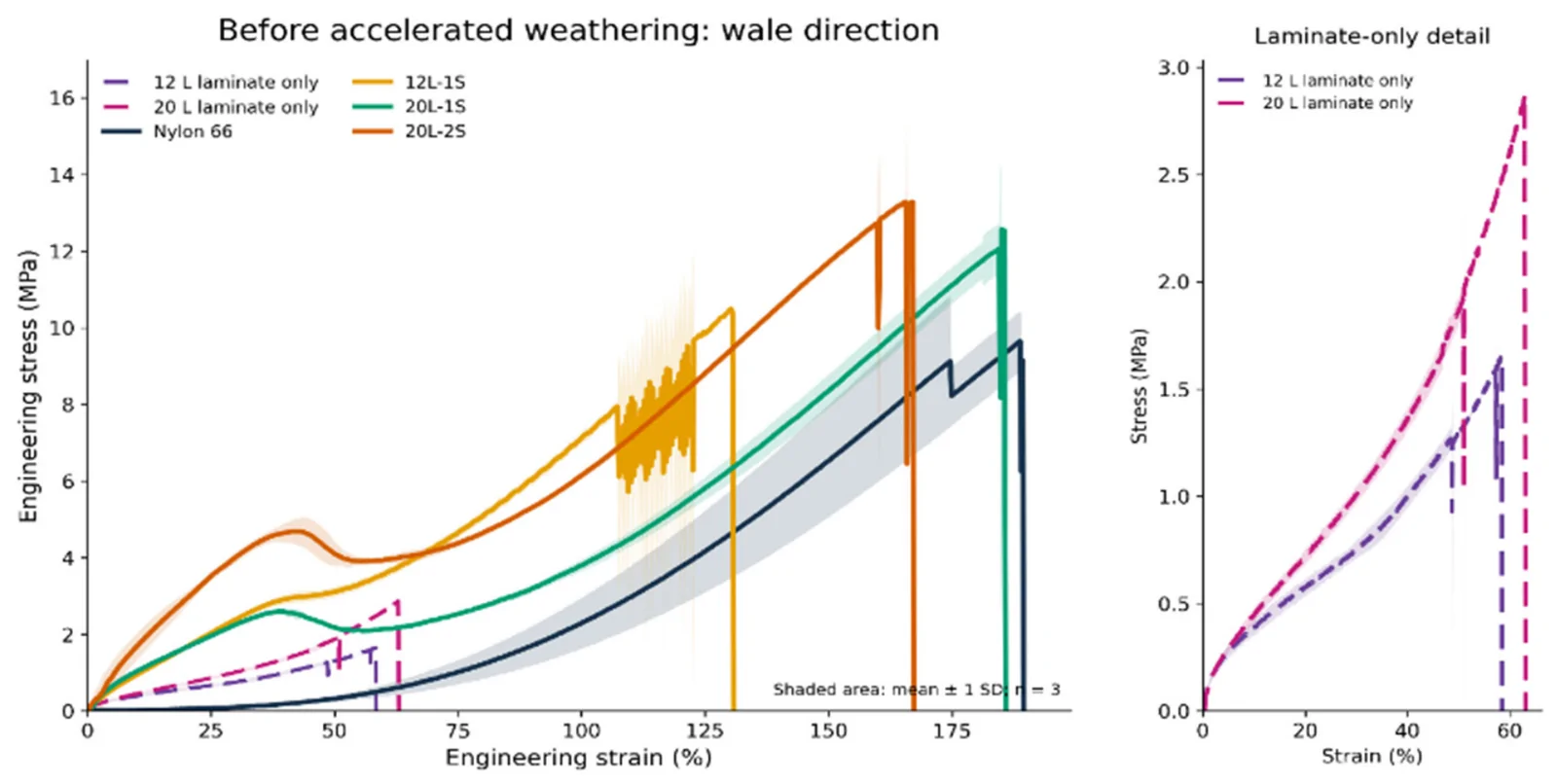
Mean stress–strain curves before weathering, wale direction (n = 3; shading = ±1 SD where ≥2 specimens remained). Inset: unsupported APR.

**Figure 13:**
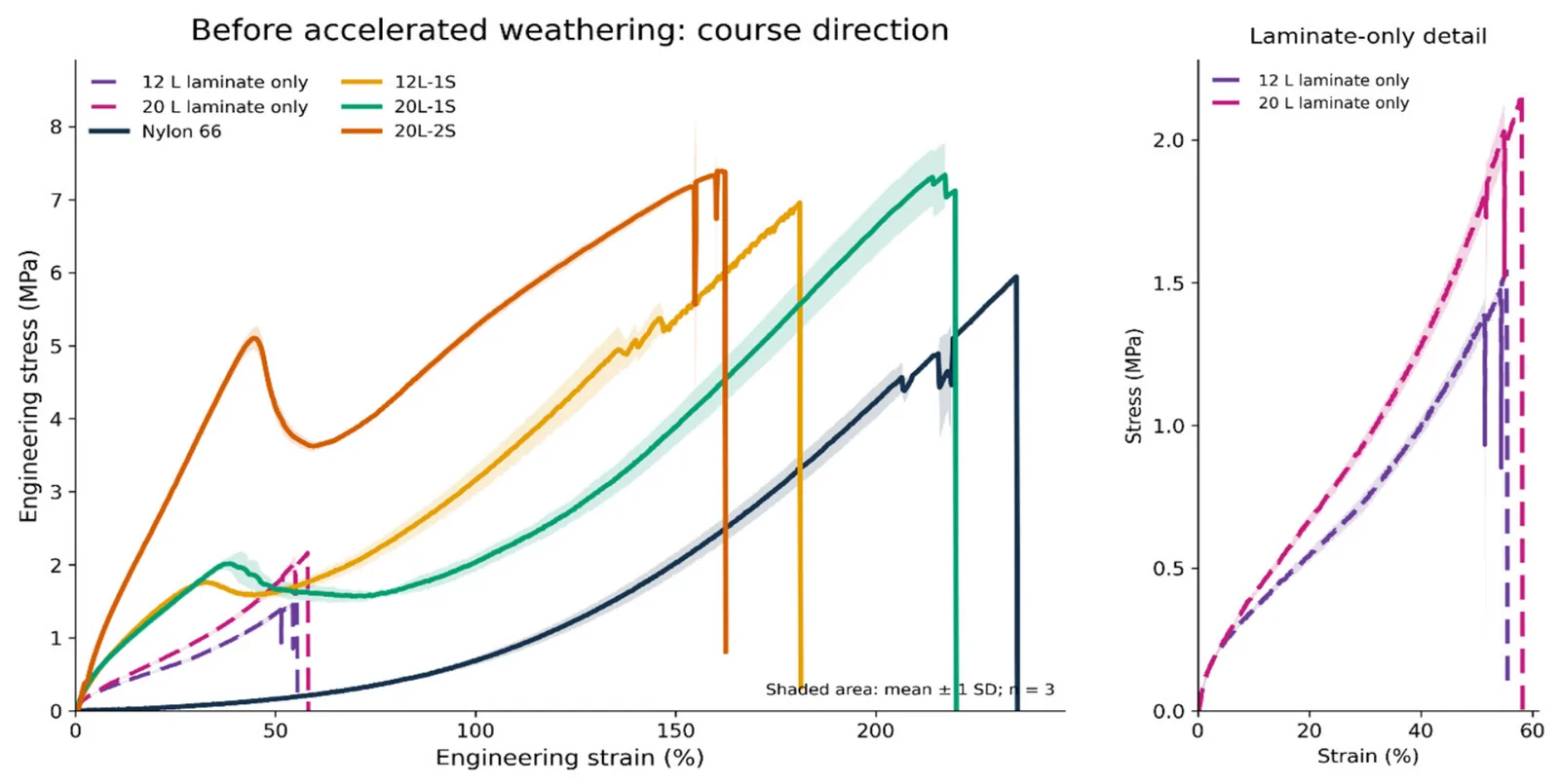
Mean stress–strain curves before weathering, course direction (n = 3; shading = ±1 SD where ≥2 specimens remained). Inset: unsupported APR.

**Figure 14:**
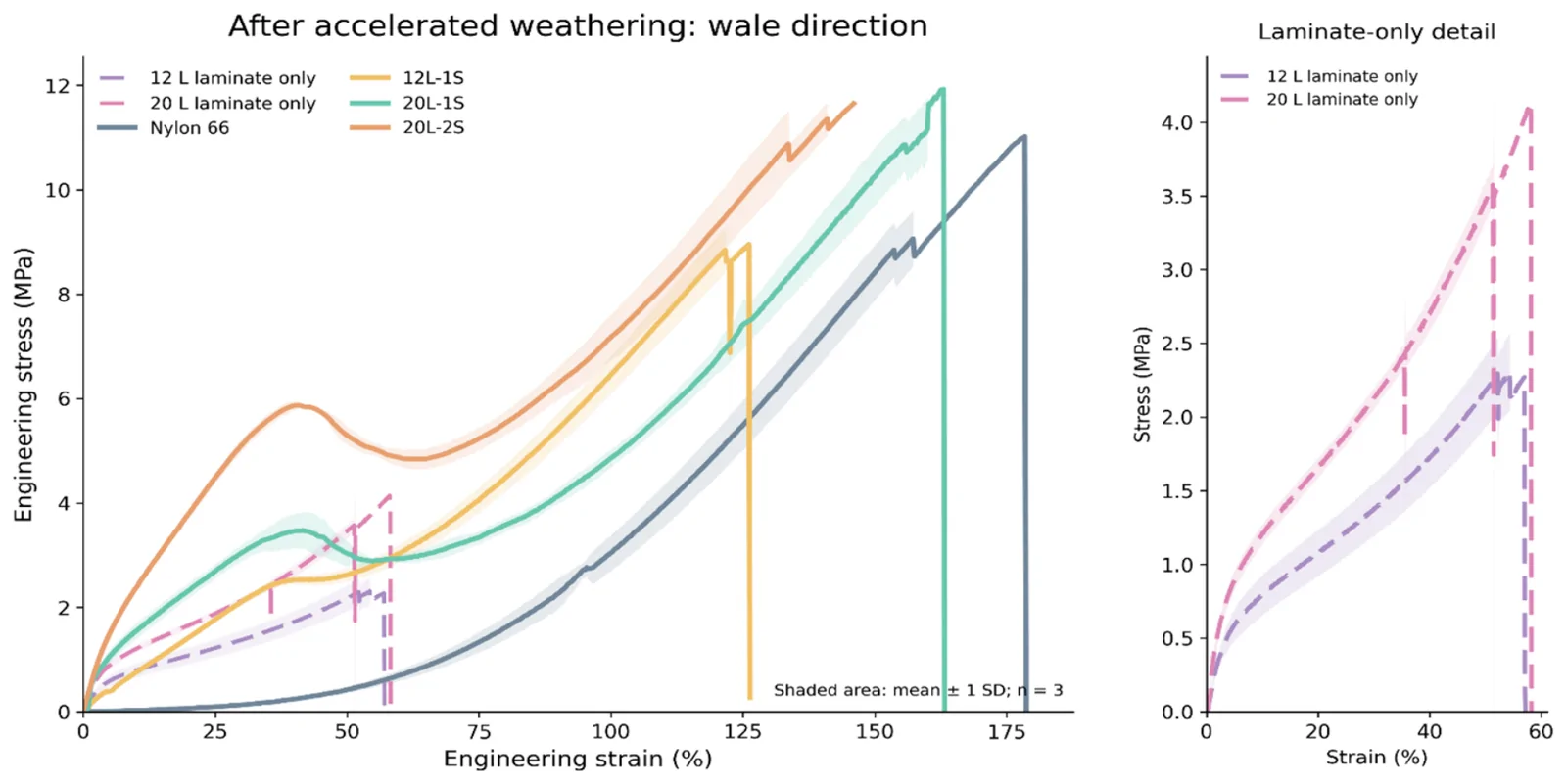
Mean stress–strain curves after 20 h weathering, wale direction (n = 3; shading = ±1 SD where ≥2 specimens remained). Inset: unsupported APR.

**Figure 15:**
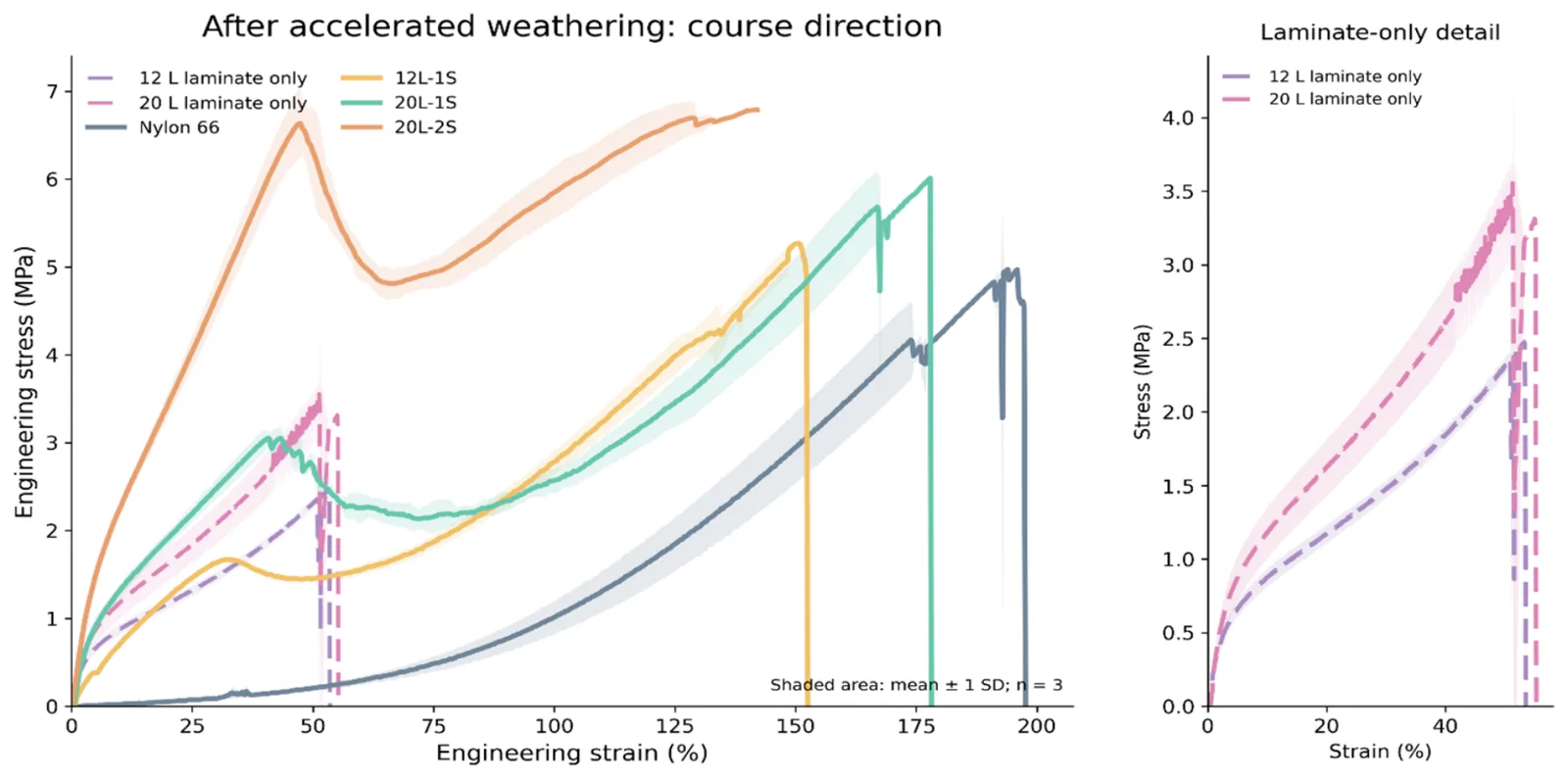
Mean stress–strain curves after 20 h weathering, course direction (n = 3; shading = ±1 SD where ≥2 specimens remained). Inset: unsupported APR.

**Figure 16:**
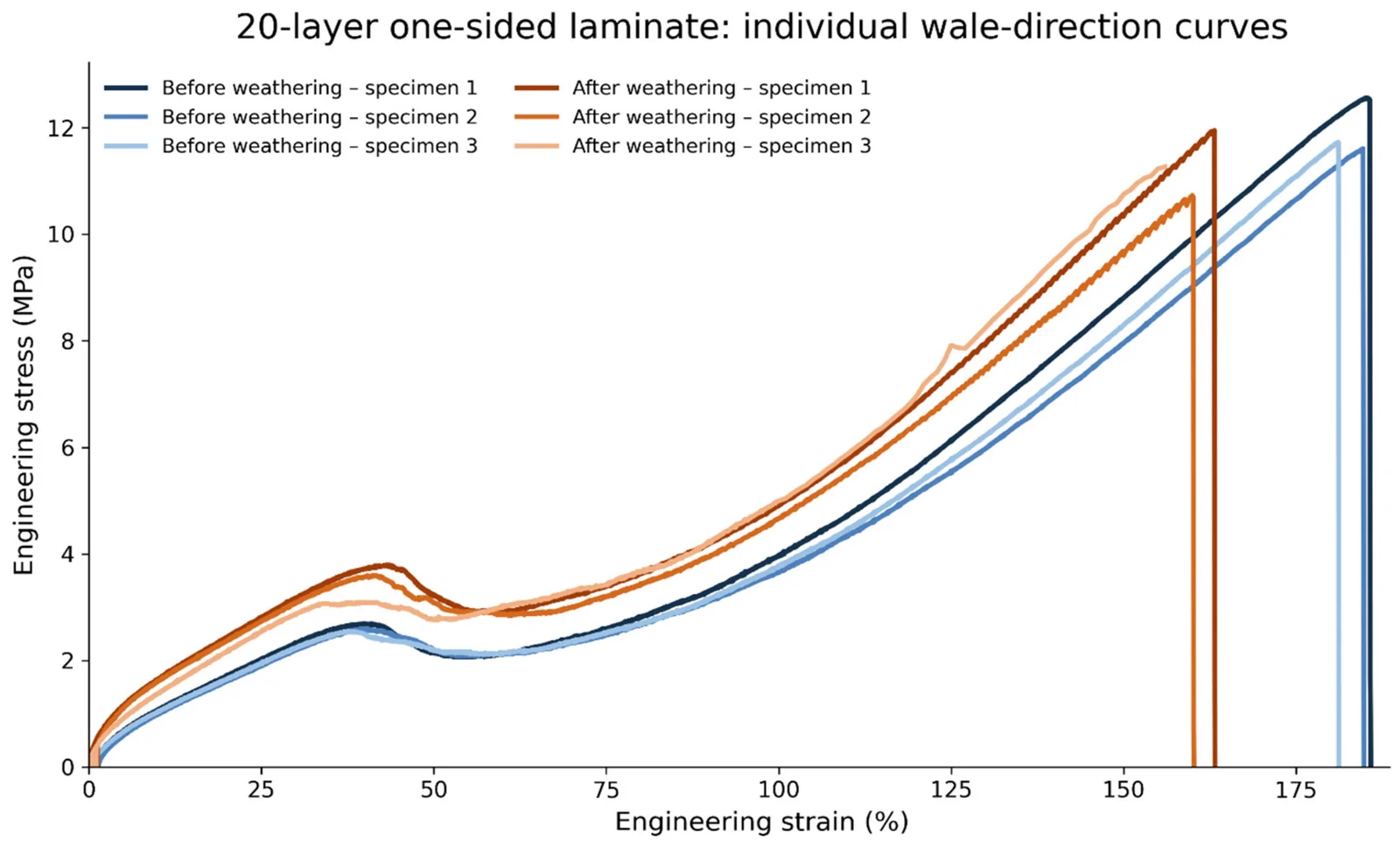
Individual 20L-1S curves, wale direction, before and after 20 h weathering (n = 3 per condition).

**Figure 17:**
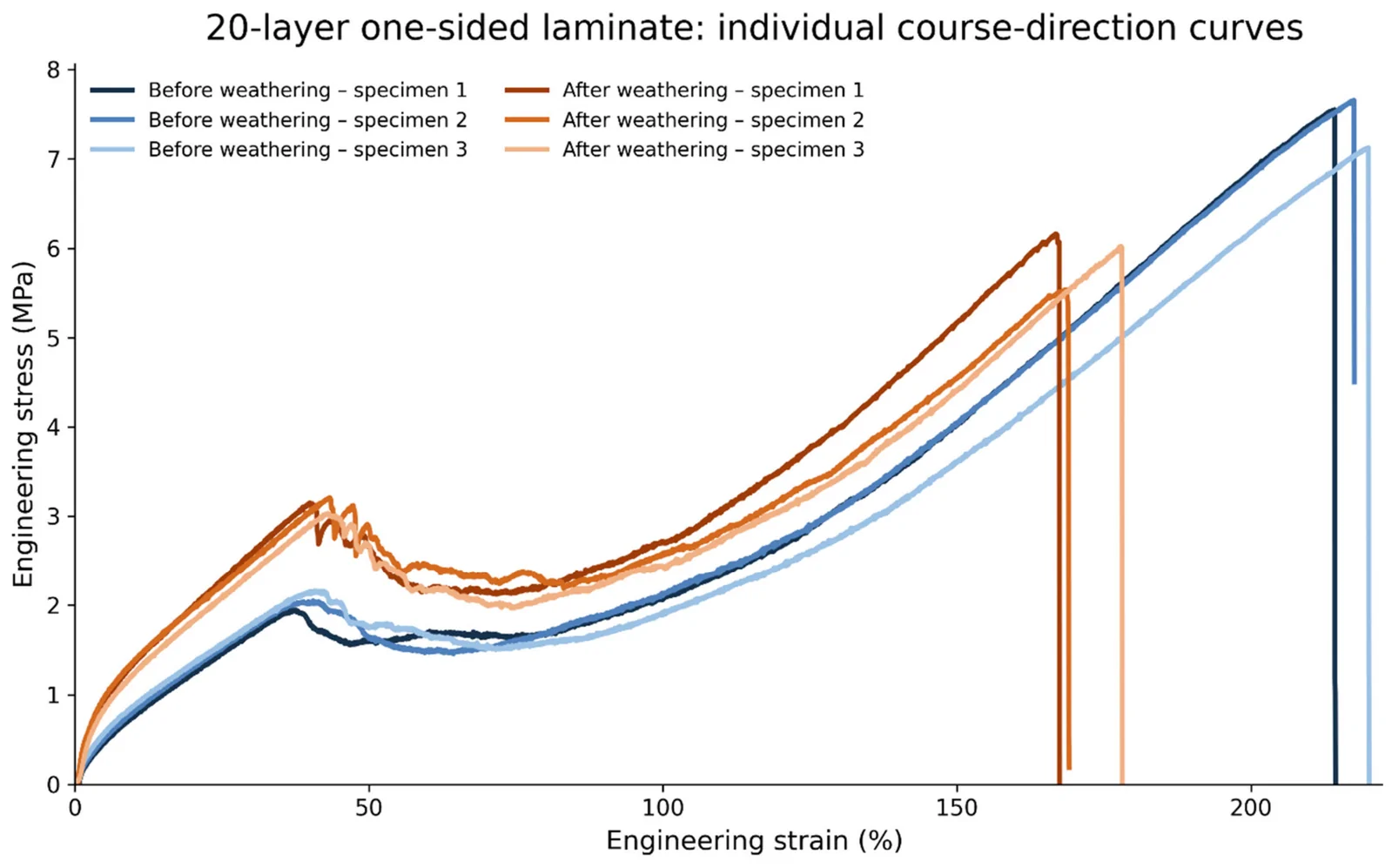
Individual 20L-1S curves, course direction, before and after 20 h weathering (n = 3 per condition).

**Figure 18:**
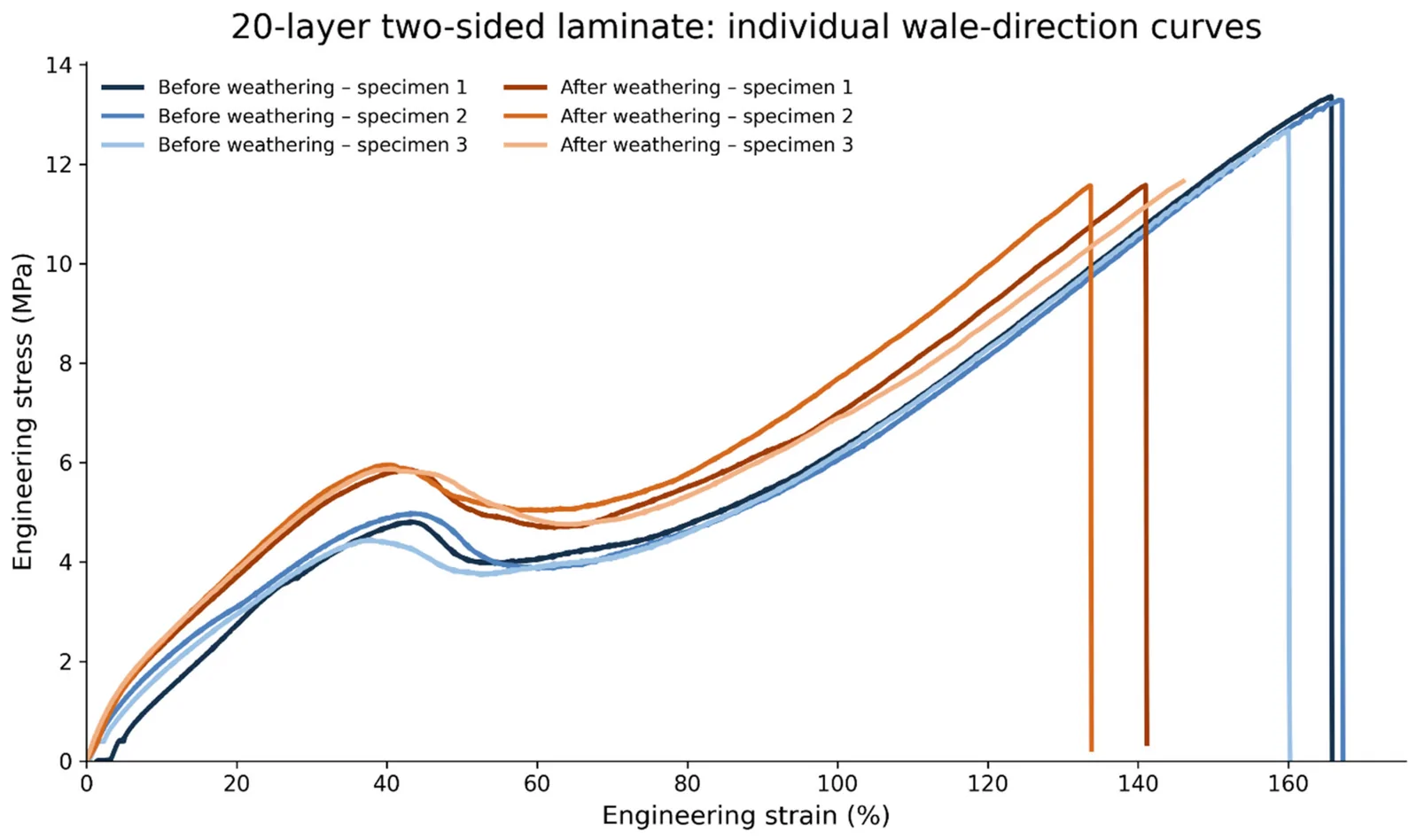
Individual 20L-2S curves, wale direction, before and after 20 h weathering (n = 3 per condition).

**Figure 19:**
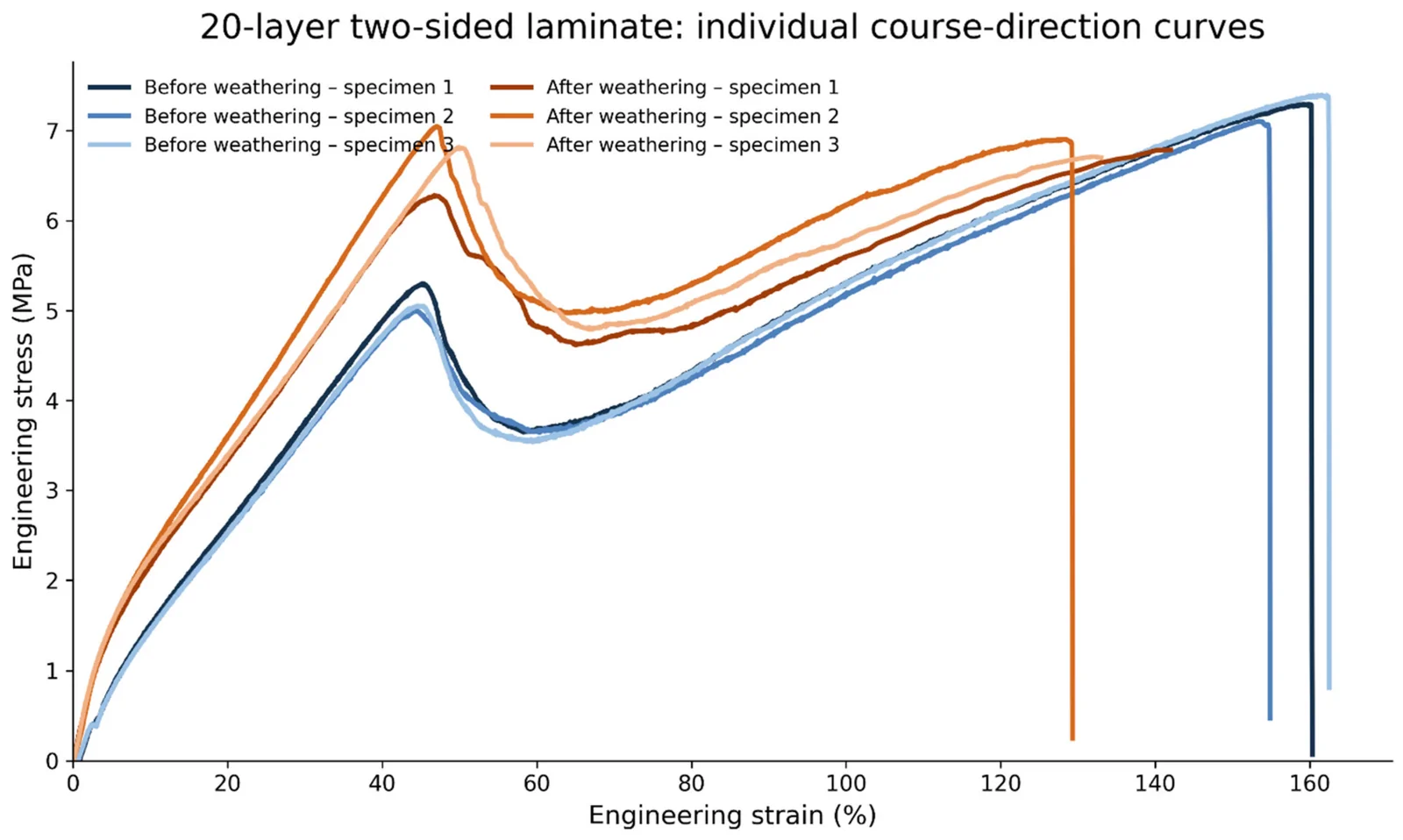
Individual 20L-2S curves, course direction, before and after 20 h weathering (n = 3 per condition).

**Figure 20:**
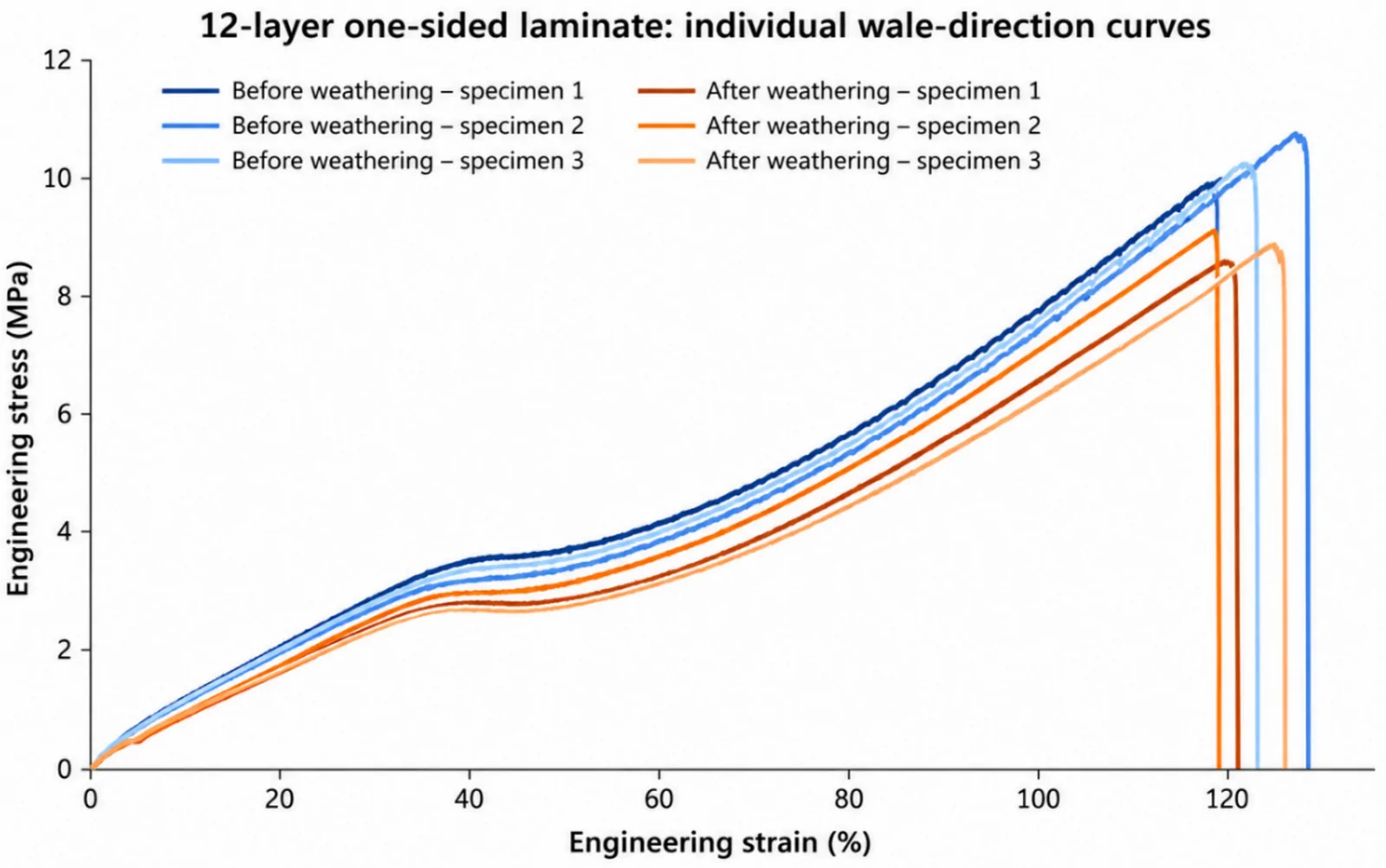
Individual 12L-1S curves, wale direction, before and after 20 h weathering (n = 3 per condition).

**Figure 21:**
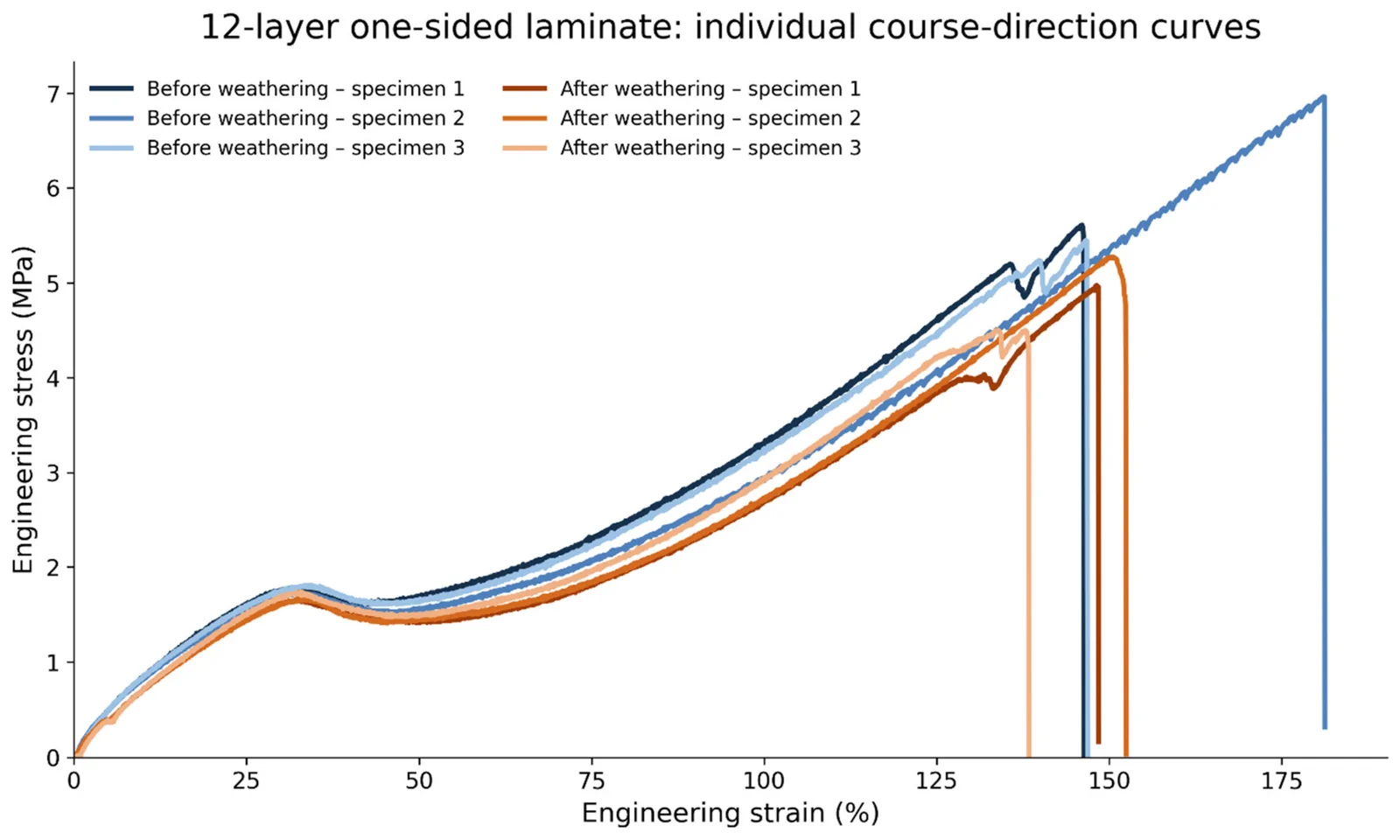
Individual 12L-1S curves, course direction, before and after 20 h weathering (n = 3 per condition).

**Figure 22:**
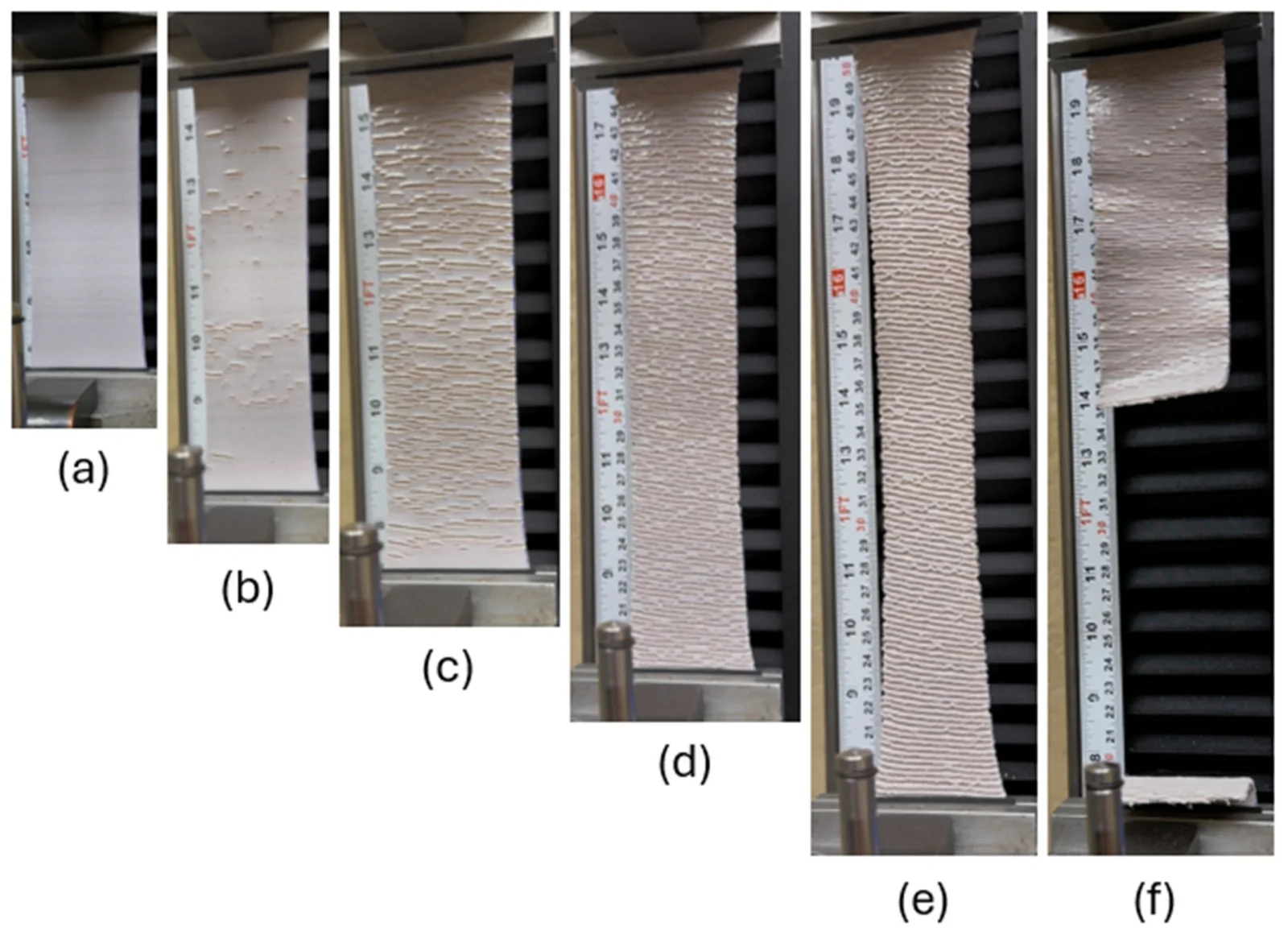
Progressive 20L-2S deformation in course-direction tension: (**a**) intact, (**b**) first APR cracks, (**c**) crack multiplication, (**d**) continued knit extension, (**e**) yarn alignment, and (**f**) final rupture.

**Figure 23:**
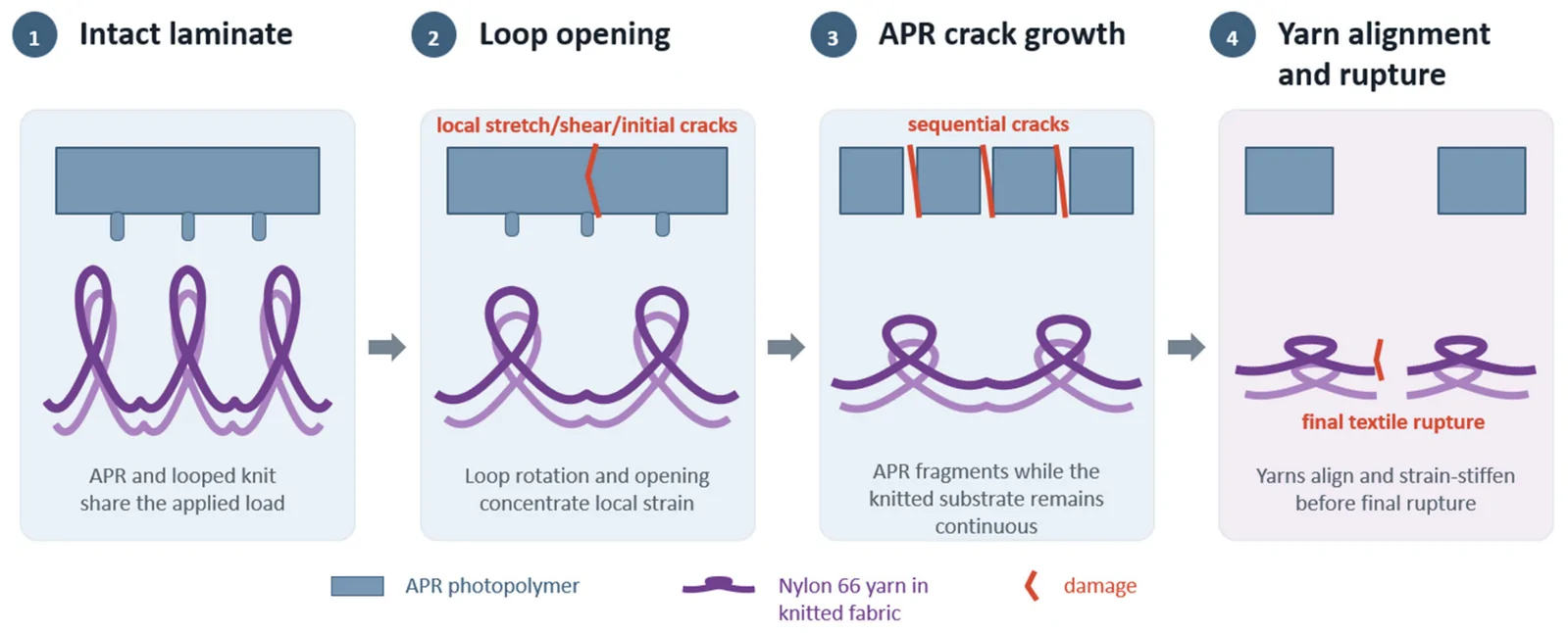
Conceptual schematic of the proposed multistage deformation and failure mechanism of the fabric-supported APR/Nylon 66 laminate during tensile loading: (1) the intact APR layer and knitted substrate initially share the applied load; (2) loop rotation and opening concentrate local bending and shear in the continuous APR layer; (3) the APR laminate is the first component to undergo structural failure through sequential cracking and fragmentation, while the knitted substrate remains continuous; and (4) progressive yarn alignment and strain stiffening precede final textile rupture. The schematic is interpretive and does not represent a direct microscale observation.

Table 3 summarizes the mean maximum engineering stress, and Table 4 summarizes the mean maximum engineering strain. Figure 12, Figure 13, Figure 14 and Figure 15 present the corresponding mean engineering stress–strain curves, Figure 16, Figure 17, Figure 18, Figure 19, Figure 20 and Figure 21 show the individual specimen curves for the fabric-supported laminates, and Figure 22 documents progressive deformation and failure of a representative 20L-2S specimen, and Figure 23 summarizes the proposed multi-stage mechanism. The effects of loading direction, printed-layer number, laminate placement, and accelerated exposure are discussed in the following sections.

Figure 13 further illustrates the strength–extensibility trade-off in the course direction: the 20L-1S architecture retained substantially greater strain capacity, whereas the 20L-2S architecture exhibited the highest maximum engineering stress.

Course-direction specimens generally reached greater strain before failure than wale-direction specimens. The knit accommodated extension through loop opening, rotation, yarn bending, and interloop rearrangement before yarn segments became aligned and directly loaded. Table 4 reports maximum engineering strain.

The fluctuations and stepwise stress drops visible in the fabric-supported curves, particularly after the first local maximum, are interpreted as sequential APR cracking or local slip followed by load redistribution to intact APR regions and the knitted substrate. The shaded-band variations also reflect specimen-to-specimen differences in the strain at which these local events occurred. Because synchronized high-speed imaging, acoustic emission, or microscopy was not used, this interpretation remains provisional rather than an event-by-event identification.

#### 3.2.1. Directional Response of Reference Nylon 66 Knit

The unprinted Nylon 66 interlock knit exhibited a strongly direction-dependent tensile response. Before accelerated weathering, the reference fabric reached a mean maximum engineering stress of 10.13 ± 0.92 MPa in the wale direction and 5.22 ± 0.63 MPa in the course direction. The corresponding maximum engineering strains were 184.40 ± 8.44% and 221.23 ± 13.33%, respectively. Thus, the wale direction exhibited greater load-bearing capacity, whereas the course direction accommodated greater deformation before final failure. After accelerated weathering, the maximum engineering stress decreased to 9.91 ± 0.97 MPa in the wale direction and 4.89 ± 0.17 MPa in the course direction, while the maximum engineering strain decreased to 163.30 ± 13.44% and 190.73 ± 11.86%, respectively. The directional difference therefore remained after exposure. This combination of higher wale-direction stress and greater course-direction strain reflects the anisotropic deformation of the knitted structure, in which loop geometry and yarn orientation govern the progression from loop rearrangement and opening to direct yarn loading.

#### 3.2.2. Effect of Laminate Architecture Before Accelerated Weathering

Before accelerated exposure, the 20-layer two-sided (20L-2S) architecture produced the highest maximum engineering stress in both loading directions, reaching 13.11 ± 0.37 MPa in the wale direction. In the course direction, the two 20-layer architectures exhibited similar maximum engineering stress values, but the 20L-2S structure showed the slightly higher mean value, reaching 7.44 ± 0.28 MPa, compared with 7.26 ± 0.15 MPa for the 20L-1S structure. The difference between the two course-direction values was small, only 0.18 MPa, and should not be interpreted as evidence of a substantial mechanical advantage of the two-sided architecture. Nevertheless, both 20-layer configurations produced a clear increase in maximum engineering stress relative to the unprinted Nylon 66 knit.

In Figure 12, Figure 13, Figure 14 and Figure 15, dashed lines represent the unsupported 3DPP laminate-only specimens; the accompanying enlarged views show these lower-stress curves separately; 12L-1S—Nylon 66 fabric reinforced with a 12-layer 3DPP laminate on one side; 20L-1S—Nylon 66 fabric reinforced with a 20-layer 3DPP laminate on one side; 20L-2S—Nylon 66 fabric reinforced with 20-layer 3DPP laminates on both sides.

Before accelerated weathering, the unprinted Nylon 66 knit exhibited the highest mean extensibility, reaching 184.40 ± 8.44% engineering strain in the wale direction and 221.23 ± 13.33% in the course direction. The 20L-1S architecture retained nearly the full extensibility of the reference fabric, reaching 183.94 ± 2.45% in the wale direction and 217.34 ± 2.88% in the course direction. In comparison, the 20L-2S architecture reached 164.42 ± 3.73% and 159.23 ± 3.93%, respectively, while the 12L-1S structure reached 126.29 ± 4.15% and 158.15 ± 19.98%. These results demonstrate a trade-off between reinforcement and extensibility: two-sided printing increased the maximum engineering stress but more strongly constrained deformation of the knitted substrate, whereas the 20-layer one-sided architecture preserved substantially greater extensibility.

#### 3.2.3. Response of Fabric-Supported Laminates After Accelerated Weathering

Accelerated xenon-arc weathering reduced the maximum engineering stress of all fabric-supported laminate architectures. The 12-layer one-sided structure decreased from 10.05 to 9.00 MPa in the wale direction and from 6.01 to 4.92 MPa in the course direction, corresponding to reductions of approximately 10.4% and 18.1%, respectively. The 20-layer one-sided structure decreased from 11.97 to 11.32 MPa in the wale direction and from 7.26 to 5.90 MPa in the course direction, corresponding to reductions of approximately 5.4% and 18.7%, respectively. The 20-layer two-sided structure decreased from 13.11 to 11.60 MPa in the wale direction and from 7.44 to 6.88 MPa in the course direction, corresponding to reductions of approximately 11.5% and 7.5%, respectively. The results indicate that the effect of accelerated weathering depended on both loading direction and laminate architecture. The greatest reduction was observed for the 20-layer one-sided structure tested in the course direction, whereas the 20-layer two-sided structure showed the smallest reduction in the course direction. After weathering, both 20-layer architectures continued to exhibit greater maximum engineering stress than the weathered unprinted Nylon 66 fabric in both directions. In contrast, the weathered 12-layer one-sided structure remained below the reference fabric in the wale direction and was nearly equal to it in the course direction. Thus, the thicker 20-layer configurations retained a clearer reinforcement effect after the 20 h exposure.

Accelerated xenon-arc exposure also reduced the maximum engineering strain of all fabric-supported laminates. For the 12L-1S structure, strain decreased from 126.29 to 123.58% in the wale direction and from 158.15 to 146.41% in the course direction, corresponding to reductions of 2.1% and 7.4%, respectively. For the 20L-1S architecture, strain decreased from 183.94 to 159.80% in the wale direction and from 217.34 to 171.52% in the course direction, corresponding to reductions of 13.1% and 21.1%. The 20L-2S architecture decreased from 164.42 to 140.30% in the wale direction and from 159.23 to 134.75% in the course direction, corresponding to reductions of 14.7% and 15.4%, respectively. Thus, the greatest loss of extensibility occurred for the 20L-1S structure in the course direction, whereas the 12L-1S structure showed the smallest strain reduction in the wale direction.

After weathering, the same strength–extensibility trade-off remained: the 20L-2S architecture retained the highest maximum engineering stress, whereas the 20L-1S architecture retained greater maximum strain in both loading directions. The corresponding mean stress–strain curves are presented in Figure 14 and Figure 15.

The 12L-1S structure generally failed at lower strains than the 20L-1S structure. This does not necessarily indicate that the thinner laminate was intrinsically more brittle. Instead, differences in laminate continuity, penetration, cracking, and load transfer may have influenced the point at which the combined textile–photopolymer structure failed. The individual course-direction curves also showed greater specimen-to-specimen variability for the 12L-1S architecture, including one unexposed specimen that extended substantially farther than the other specimens.

Accelerated xenon-arc exposure generally reduced the strain sustained by the fabric-supported structures before final rupture. This reduction was particularly evident for the 20L-1S laminate in both directions and for the 20L-2S laminate in the wale direction. The change was smaller for the 12L-1S laminate in the wale direction, whereas the course-direction results showed greater variability. When considered together with the reductions in maximum engineering stress, the lower failure strains indicate that accelerated exposure reduced the ability of the fabric-supported laminates to sustain large deformation.

The unsupported laminate-only specimens remained substantially less extensible than the fabric-supported structures and generally failed at approximately 50–60% engineering strain. Although their maximum engineering stress increased after accelerated exposure, the curves did not show a corresponding increase in extensibility. This combination is consistent with exposure-induced stiffening or additional curing; however, whether the exposure also caused embrittlement cannot be established from maximum stress and strain alone. The strain response also clarifies the staged failure mechanism of the fabric-supported laminates. The initial shoulder or local reduction in stress, generally occurring between approximately 35% and 60% strain, was associated with cracking, fragmentation, or local separation of the APR layer. However, this event did not represent complete specimen failure. The Nylon 66 knitted substrate remained continuous and accommodated substantial additional deformation before the yarns became aligned and taut, producing renewed strain stiffening and eventual rupture. Consequently, the high strain at final failure primarily reflects the continued load-bearing capacity of the knitted substrate after damage to the 3DPP laminate.

#### 3.2.4. Laminates Without Nylon 66 Fabric

The unsupported laminate-only specimens exhibited substantially lower maximum stresses than the fabric-supported structures but showed the opposite exposure-related trend. The 12-layer laminate-only specimens increased from 1.52 ± 0.17 to 2.43 ± 0.13 MPa in the wale-related orientation and from 1.48 ± 0.05 to 2.47 ± 0.02 MPa in the course-related orientation. The 20-layer laminate-only specimens increased from 2.13 ± 0.67 to 3.41 ± 0.89 MPa and from 2.02 ± 0.20 to 3.58 ± 0.01 MPa, respectively. These changes corresponded to increases of approximately 59.9–77.2%.

The increased maximum stress of the unsupported photopolymer after exposure was consistent with additional curing or exposure-induced stiffening. Additional photon exposure can increase conversion and crosslink density in incompletely cured photopolymers, raising stiffness and strength, although extended exposure can also promote embrittlement [34,35,36]. However, maximum stress alone cannot distinguish beneficial post-curing from embrittlement. The result should therefore be considered together with changes in strain at failure, curve shape, visible cracking, and surface condition.

Despite the substantial increase in maximum stress after exposure, the unsupported laminates did not exhibit a corresponding increase in extensibility. The maximum strain of the 12-layer laminate changed only from 54.80 ± 5.32% to 54.61 ± 2.41% in the wale-related orientation and from 53.73 ± 2.11% to 52.20 ± 1.15% in the course-related orientation. For the 20-layer laminate, strain decreased from 53.60 ± 8.41% to 48.39 ± 11.62% and from 54.94 ± 3.19% to 52.82 ± 2.13%, respectively. Thus, accelerated exposure increased the maximum stress of the unsupported APR without increasing its strain capacity, supporting an interpretation of exposure-induced stiffening or additional curing.

#### 3.2.5. Nonlinear Behavior

The stress–strain curves demonstrated that maximum stress represented only one aspect of the mechanical response. The fabric-supported laminates exhibited strongly nonlinear behavior characterized by an initial increase in stress, a shoulder or local stress reduction, and subsequent strain stiffening. This response was particularly evident in the 20-layer one-sided and two-sided structures. The first local maximum was consistent with the onset of cracking, fragmentation, or local separation of the printed laminate. Following this event, the knitted Nylon 66 substrate remained intact and continued to deform through loop rotation, loop opening, yarn bending, and interloop rearrangement. At higher strain, the yarn segments became progressively aligned and taut, producing renewed strain stiffening before final rupture. The resulting high-strain response was hyperelastic-like in shape; however, the complete textile–photopolymer structure should not be described as purely hyperelastic because the laminate underwent irreversible damage and elastic recovery was not evaluated through unloading–reloading testing. Individual stress–strain curves for the 20L-1S, 20L-2S, and 12L-1S architectures in both loading directions are presented in Figure 16, Figure 17, Figure 18, Figure 19, Figure 20 and Figure 21. The progressive cracking and continued deformation of a representative 20L-2S specimen are illustrated in Figure 22, whereas Figure 23 presents a conceptual schematic of the proposed multistage deformation and failure mechanism during tensile loading.

## 4. Discussion

The tensile behavior of the textile–APR structures was governed by interaction between a comparatively continuous APR surface and a deformable Nylon 66 knit. SEM showed localized APR entry into inter-yarn and inter-filament spaces. This morphology supports a hypothesis of geometric engagement, but the global strip tests do not quantify interface strength. Peel or interfacial-shear testing is therefore required before claims can be made about resin-to-fabric bond strength, and post-tensile cross-sectional SEM is required to distinguish delamination, yarn pull-out, and APR matrix fracture at the microscale.

Laminate architecture produced a clear trade-off between reinforcement and extensibility. The 20-layer two-sided (20L-2S) architecture exhibited the highest maximum engineering stress in both loading directions before accelerated weathering, reaching 13.11 ± 0.37 MPa in the wale direction and 7.44 ± 0.28 MPa in the course direction. In contrast, the 20-layer one-sided (20L-1S) architecture retained substantially greater extensibility, reaching 183.94 ± 2.45% strain in the wale direction and 217.34 ± 2.88% in the course direction, compared with 164.42 ± 3.73% and 159.23 ± 3.93%, respectively, for the two-sided structure. Thus, increasing the amount of APR and constraining both surfaces of the knit increased load-bearing capacity but restricted the loop-mediated deformation that normally allows knitted fabrics to sustain very large strains. The 20L-2S configuration can therefore be considered the strongest architecture based on maximum engineering stress, whereas the 20L-1S configuration provided a more favorable balance between reinforcement and extensibility.

The nonlinear stress–strain response further illustrates the different roles of the laminate and textile substrate. In the fabric-supported structures, an initial shoulder or local stress reduction generally occurred at approximately 35–60% strain and was associated with cracking, fragmentation, or local separation of the APR (see Figure 22b,c). This did not represent complete failure of the specimen. The knitted Nylon 66 remained continuous and accommodated substantial additional deformation before the response strain-stiffened as yarns aligned (Figure 22d and Figure 23), ultimately leading to rupture. The high-strain response was therefore hyperelastic-like in shape, but the complete laminate should not be described as purely hyperelastic because irreversible cracking of the APR occurred and elastic recovery was not evaluated. This staged failure behavior is important for potential technical applications because damage to the printed surface did not immediately eliminate the load-bearing capability of the textile substrate.

The directional response is related to both loop topology and density. The substrate contained 124 wales and 150 courses per 10 cm, corresponding to nominal repeat spacings of approximately 0.81 and 0.67 mm, respectively. Density alone does not determine failure because yarn path and loop orientation govern how these repeats rotate and open under load. Under course-direction loading, curved loop segments can open and rearrange over a longer strain range, consistent with the greater measured extensibility. Wale-direction loading brings loop legs into the load path earlier, producing higher stress at lower strain. Because the continuous APR bridges these periodically deforming cells, local bending and shear can concentrate at resin-filled pores and filament-contact regions, providing plausible crack-initiation sites in the 35–60% strain range. The higher course count supplies more repeated contact sites per unit length and may distribute multiple local events, but the present experiment does not isolate density from topology.

The curve shapes also extend prior direct-to-textile studies. Published polyjet and material-extrusion work has largely emphasized adhesion force, peel resistance, surface morphology, or localized printed features [13,14,18,19,20,21,22,23]. Those studies show that printing conditions and textile openness affect penetration and adhesion, while knit-based composites demonstrate that loop geometry changes deformation and energy absorption [13,14,23]. The present curves add the global large-strain response of a continuous polyjet surface on an interlock knit: APR damage occurs well before final textile rupture, and one- versus two-sided printing shifts the balance between maximum stress and extensibility. Numerical stress values should not be compared directly with reported peel forces or bending data because the loading modes, stress definitions, materials, and geometries differ.

One of the most unexpected findings was the response of the unsupported APR laminates to accelerated xenon-arc exposure (laminates only, no fabric). Whereas the fabric-supported laminates generally showed reductions in maximum engineering stress after exposure, the unsupported APR specimens became considerably stronger. Maximum engineering stress increased by approximately 59.9–77.2%, depending on laminate thickness and test/sample orientation. At the same time, their maximum strain remained similar or decreased slightly rather than increasing. Additional photon exposure can increase conversion and crosslink density in incompletely cured photopolymers, raising stiffness and strength, although extended exposure may also promote embrittlement [37]. The present results are therefore consistent with post-curing or exposure-induced stiffening, but tensile data alone cannot distinguish increased conversion from oxidation, physical aging, or other structural changes. FTIR, degree-of-conversion analysis, DSC, DMA, hardness, and fracture-surface characterization are required to test this mechanism.

The behavior of the complete textile–APR laminates was different because xenon exposure affected not only the APR but also the Nylon 66 substrate and potentially the interface between them. The unprinted Nylon 66 reference retained most of its maximum engineering stress after exposure but showed a noticeable decrease in extensibility: maximum strain decreased by 11.4% in the wale direction and 13.8% in the course direction.

Recent xenon-arc work on Nylon 6,6 webbings likewise demonstrates exposure-related tensile-strength loss and the value of chemical characterization when surface morphology changes are limited [38].

In the combined structure, reduced Nylon 66 extensibility could restrict loop-mediated stress redistribution while a stiffer APR increases the stiffness mismatch between the surface layer and substrate. The resulting bending and shear concentrations at resin-filled pores and partially enclosed filaments could accelerate APR cracking or local separation. UV damage within Nylon 66, APR stiffening, and changes in the contact zone are therefore competing, not mutually exclusive, explanations. Because no peel test, post-tensile SEM, or chemical analysis was performed, the data do not establish that UV radiation specifically embrittled the interlocking zone or identify chain scission in Nylon 66. Visual observations provide additional evidence that xenon exposure altered the APR. Although quantitative color measurements were not performed, fading of the grey laminate was visually observed following accelerated exposure. The specimens were also examined for discoloration, yellowing, surface cracking, laminate damage, and local separation of the printed layer from the textile, as specified in the experimental procedure. Because color change was not measured instrumentally, the observed fading should be described qualitatively rather than interpreted as a quantified photodegradation response. Nevertheless, the visible fading demonstrates that the xenon exposure produced an observable change in the printed material and supports the need for future colorimetric and chemical characterization of the APR following environmental exposure.

The surface morphology of the large-area laminates also highlights the importance of the manufacturing process. Parallel striations were visible across the printed panels and were attributed to the jetting and curing path used during APR deposition. SEM examination confirmed corresponding surface undulations. These features indicate that large-area polyjet deposition did not produce a perfectly uniform surface, even though the laminate was macroscopically continuous. Variations in deposition path, layer overlap, curing conditions, and processing speed could potentially influence surface uniformity and local laminate thickness. In particular, faster production may reduce the time available for uniform droplet deposition, leveling, or curing and could therefore increase process-related surface irregularities. However, printing speed was not varied systematically in the present study, so a direct relationship between production speed and striation formation cannot be established from these results. This should therefore be considered a hypothesis for future process-optimization studies rather than a conclusion of the present work.

The presence of striations may also have mechanical implications. Local differences in surface profile and laminate thickness could influence stiffness, crack initiation, and stress distribution during tensile loading.

Overall, the results show that continuous large-area 3DPP on knitted textiles produces a mechanically complex laminate in which the advantages of the two constituents are not simply additive. The APR provides reinforcement and surface continuity, whereas the knitted Nylon 66 provides the large-strain deformation capacity characteristic of loop-based textile structures. Two-sided 20-layer printing maximized maximum engineering stress but constrained extensibility, while one-sided 20-layer printing preserved substantially more of the deformability of the original knit. Accelerated xenon exposure further demonstrated that the constituents can respond in opposite ways: the unsupported APR became stronger and apparently stiffer, whereas the Nylon 66 lost extensibility and the complete textile-supported structures showed reduced tensile performance. These findings emphasize that optimization of 3DPP technical textiles requires consideration not only of polymer properties and printed-layer number but also of textile architecture, interfacial load transfer, environmental exposure, and large-area printing conditions.

## 5. Conclusions

Continuous large-area 3D polyjet-printed APR laminates were successfully produced directly on Nylon 66 interlock-knitted fabric, forming textile–photopolymer structures measuring approximately 330 × 432 mm. SEM observations showed that the APR formed a continuous external laminate and penetrated locally between yarns and individual filaments, indicating close geometric engagement; however, interface strength was not measured.

The tensile response was strongly dependent on knitted-fabric direction and laminate architecture. The unprinted Nylon 66 exhibited higher maximum engineering stress in the wale direction but greater extensibility in the course direction. Incorporation of the APR increased the load-bearing capacity of the knitted structure, particularly for the 20-layer configurations. The 20-layer two-sided architecture produced the highest maximum engineering stress before accelerated weathering, reaching 13.11 ± 0.37 MPa in the wale direction and 7.44 ± 0.28 MPa in the course direction. In contrast, the 20-layer one-sided architecture retained substantially greater extensibility, reaching 183.94 ± 2.45% and 217.34 ± 2.88% engineering strain in the wale and course directions, respectively. Consequently, two-sided printing maximized strength, whereas one-sided 20-layer printing provided a more favorable balance between reinforcement and preservation of knitted-fabric extensibility.

Accelerated xenon-arc weathering produced contrasting constituent responses. Unsupported APR maximum stress increased by 59.9–77.2% without increased strain capacity, whereas unprinted Nylon 66 lost extensibility and all fabric-supported laminates lost maximum stress and strain. APR post-curing or stiffening, Nylon 66 degradation, and increased constituent mismatch are plausible joint mechanisms; they cannot be separated without chemical, thermal, and interface-specific measurements. The fabric-supported laminates exhibited strongly nonlinear, staged tensile failure. Cracking or fragmentation of the APR occurred before final specimen rupture, while the underlying knitted substrate remained continuous and continued to deform to much greater strains. This behavior demonstrates that the textile substrate retained a load-bearing role after initial laminate damage.

Overall, the study demonstrates that continuous 3DPP can be used to engineer large-area textile–photopolymer laminates with tunable mechanical behavior. Increasing laminate coverage enhances load-bearing capacity but can reduce the large-strain deformability characteristic of knitted textiles. The observed strength–extensibility trade-off and the different weathering responses of the polymer and textile components should therefore be considered when designing 3DPP laminates for flexible barriers, protective structures, covers, and future inflatable textile systems.

## 6. Future Work

Future studies should evaluate a broader range of printed-layer counts to determine more precisely the minimum number of deposited layers required for complete pore closure and to establish how increasing laminate thickness affects flexibility, tensile performance, abrasion resistance, durability, and barrier properties. The experimental matrix should also include knitted, woven, and nonwoven substrates with different fiber types, surface roughness, porosity, extensibility, and structural stability. Such comparisons would clarify how textile architecture controls resin penetration, mechanical interlocking, interfacial bonding, and subsequent deformation.

Resin penetration should be quantified using polished, normal-to-surface cross-sections or three-dimensional imaging, with repeated measurements reported as a percentage of fabric thickness. Interface performance should be measured directly by an appropriate peel or interfacial-shear method with specimen geometry adapted to the continuous flexible laminate. Post-tensile SEM should examine identified fracture zones to distinguish APR matrix cracking, local separation, filament enclosure, and yarn pull-out.

Additional APR formulations should be compared for stiffness, toughness, and environmental resistance. Long-term durability studies should include abrasion, repeated flexing, fatigue, hydrolysis, laundering, and cyclic loading. FTIR or Raman spectroscopy, degree-of-conversion measurements, DSC, DMA, hardness, colorimetry, and fracture analysis before and after exposure would help distinguish post-curing from oxidation, embrittlement, and substrate degradation.

A particularly important direction is the development of continuous 3DPP textile laminates for inflatable and pressure-bearing textile structures. Although the 12-layer configuration produced a visually continuous surface without apparent open pores, visual pore closure alone does not demonstrate airtightness, waterproofness, or pressure retention. Future work should therefore include air-permeability, hydrostatic-pressure, leakage, pressure-retention, and seam-integrity testing. Burst-strength and cyclic inflation–deflation experiments should be performed to determine whether the printed laminate can withstand repeated internal pressurization without cracking, delamination, or progressive loss of pressure. The effects of folding, bending, and local crease formation should also be examined because inflatable products are commonly stored in a collapsed state and repeatedly deployed.

For inflatable applications, the performance of one-sided and two-sided printing should be compared directly. A one-sided laminate may reduce mass and preserve flexibility, whereas a two-sided architecture may provide greater reinforcement, improved surface enclosure, and increased resistance to local damage. Future studies should also evaluate printed repair patches, locally reinforced high-stress regions, and graded laminate thicknesses around valves, seams, folds, and attachment points. These investigations would support the development of lightweight inflatable shelters, pressure-supported structures, flotation devices, protective cushions, flexible bladders, and other textile-based inflatable systems.

Together, these studies would establish whether continuous textile–photopolymer laminates can be developed for inflatables, flexible barriers, protective covers, waterproof structures, and other technical-textile applications.

## Figures and Tables

**Table 1:** Experimental architectures and placement of APR layers on the technical face and back.

Sample	100% Nylon 66 Interlock Stitch, Weft Knitted Fabric	3DPP Lamination on		Note
		The Face of the Fabrics	The Back of the Fabric	
1	Yes	-		Reference fabric/no 3DPP
2	-	12 layers		Reference 3DPP/no fabric
3	-	20 layers		Reference 3DPP/no fabric
4	Yes	12 layers	-	-
5	Yes	12 layers	12 layers	Produced samples were damaged during tensile testing (grip failure and slippage); thus, some of the results were not reported
6	Yes	20 layers	-	-
7	Yes	20 layers	20 layers	-

**Table 2:** Physical characteristics of the Nylon 66 knit, unsupported APR, and fabric-supported laminates (mean ± SD; n = 10).

Material	Weight [g/m^2^] ±SD	Thickness [mm] ±SD	Number of Wales/10 cm	Number of Courses/10 cm
Nylon 66	223 ± 4.5	0.940 ±0.020	124	150
12 layers-laminate only	386.5 ± 6.2	0.2540 ± 0.050	n/a	n/a
20 layers-laminate only	569.6 ± 6.5	0.5160 ± 0.010	n/a	n/a
12 layers-one side print on Nylon 66	797 ± 7.5	1.1940 ± 0.020	n/a	n/a
12 layers—two-side print on Nylon 66	1168 ± 5.5	1.470 ± 0.030	n/a	n/a
20 layer—one-side print on Nylon 66	1010 ± 6.4	1.460 ± 0.030	n/a	n/a
20 layers—two-side print on Nylon 66	1790 ± 6.8	1.9570 ± 0.050	n/a	n/a

**Table 3:** Maximum engineering stress before and after weathering (mean ± SD; n = 3). Unsupported-APR orientations follow the surface striations; the wale-related orientation is parallel to the striations.

Material Type	Direction of Specimens	Before Accelerated Weathering; Maximum Engineering Stress [MPa]	After Accelerated Weathering; Maximum Engineering Stress [MPa]	Change in Engineering Stress: Before vs. After [%]
Nylon 66; only fabric	Wale	10.13 ± 0.92	9.91 ± 0.97	−2.2
Nylon 66; only fabric	Course	5.22 ± 0.63	4.89 ± 0.17	−6.3
12L-1S	Wale	10.05 ± 0.39	9.00 ± 0.29	−10.4
12L-1S	Course	6.01 ± 0.83	4.92 ± 0.39	−18.1
20L-1S	Wale	11.97 ± 0.52	11.32 ± 0.61	−5.4
20L-1S	Course	7.26 ± 0.15	5.90 ± 0.33	−18.7
20L-2S	Wale	13.11 ± 0.37	11.60 ± 0.05	−11.5
20L-2S	Course	7.44 ± 0.28	6.88 ± 0.14	−7.5
12 L; only laminate	Wale orientation	1.52 ± 0.17	2.43 ± 0.13	+59.9
12 L; only laminate	Course orientation	1.48 ± 0.05	2.47 ± 0.02	+66.9
20 L; only laminate	Wale orientation	2.13 ± 0.67	3.41 ± 0.89	+60.1
20 L; only laminate	Course orientation	2.02 ± 0.20	3.58 ± 0.01	+77.2

**Table 4:** Maximum engineering strain before and after weathering (mean ± SD; n = 3). Unsupported-APR orientations follow the surface striations; the wale-related orientation is parallel to the striations.

Material Type	Direction of Specimens	Before Accelerated Weathering; Maximum Engineering Strain [%]	After Accelerated Weathering; Maximum Engineering Strain [%]	Change in Engineering Strain: Before vs. After [%]
Nylon 66	Wale	184.40 ± 8.44	163.30 ± 13.44	−11.4
Nylon 66	Course	221.23 ± 13.33	190.73 ± 11.86	−13.8
12L-1S	Wale	126.29 ± 4.15	123.58 ± 2.40	−2.1
12L-1S	Course	158.15 ± 19.98	146.41 ± 7.26	−7.4
20L-1S	Wale	183.94 ± 2.45	159.80 ± 3.63	−13.1
20L-1S	Course	217.34 ± 2.88	171.52 ± 5.74	−21.1
20L-2S	Wale	164.42 ± 3.73	140.30 ± 6.16	−14.7
20L-2S	Course	159.23 ± 3.93	134.75 ± 6.55	−15.4
12L laminate only	Wale orientation	54.80 ± 5.32	54.61 ± 2.41	−0.4
12L laminate only	Course orientation	53.73 ± 2.11	52.20 ± 1.15	−2.9
20L laminate only	Wale orientation	53.60 ± 8.41	48.39 ± 11.62	−9.7
20L laminate only	Course orientation	54.94 ± 3.19	52.82 ± 2.13	−3.8

## Data Availability

The original contributions presented in this study are included in the article/Supplementary Materials. Further inquiries can be directed to the author.

## Supplementary Materials

The supporting information can be downloaded at https://doi.org/10.6084/m9.figshare.33201465, accessed on 29 August 2026. Figures S1–S5 and S8–S10 show SEM images of cross-sections of a one-sided laminate with 12 layers of 3DPP. Figures S6 and S7 show SEM images of the surface of the same one-sided laminate.
